# Temporal regulation of gene expression during auxin-triggered crown root formation in barley: an integrated approach

**DOI:** 10.1093/pcp/pcaf077

**Published:** 2025-07-13

**Authors:** Nikola Kořínková, Alexie Techer, Maria Majeská Čudejková, Dieu Thu Nguyen, David Kopečný, Bruno Contreras-Moreira, Pavla Navrátilová, Pascal Gantet, Véronique Bergougnoux

**Affiliations:** Czech Advanced Technology and Research Institute (CATRIN), Palacký University Olomouc, Šlechtitelů 241/27, Olomouc 779 00, Czech Republic; Department of Biochemistry, Faculty of Science, Palacký University Olomouc, Šlechtitelů 241/27, Olomouc 779 00, Czech Republic; Czech Advanced Technology and Research Institute (CATRIN), Palacký University Olomouc, Šlechtitelů 241/27, Olomouc 779 00, Czech Republic; Czech Advanced Technology and Research Institute (CATRIN), Palacký University Olomouc, Šlechtitelů 241/27, Olomouc 779 00, Czech Republic; Czech Advanced Technology and Research Institute (CATRIN), Palacký University Olomouc, Šlechtitelů 241/27, Olomouc 779 00, Czech Republic; Department of Experimental Biology, Faculty of Science, Palacký University Olomouc, 17. listopadu 1192/12, Olomouc 779 00, Czech Republic; Computational and Structural Biology, EEAD-CSIC, Av. Montañana 1.005, Zaragoza 50059, Spain; Centre of Plant Structural and Functional Genomics, Institute of Experimental Botany, Academy of Sciences, Šlechtitelů 893/31, Olomouc 779 00, Czech Republic; Czech Advanced Technology and Research Institute (CATRIN), Palacký University Olomouc, Šlechtitelů 241/27, Olomouc 779 00, Czech Republic; UMR DIADE, Université de Montpellier, IRD, CIRAD, Avenue Agropolis 911, Montpellier 34398, France; Czech Advanced Technology and Research Institute (CATRIN), Palacký University Olomouc, Šlechtitelů 241/27, Olomouc 779 00, Czech Republic

**Keywords:** ATAC-seq, auxin, Barley, crown roots, DAP-seq, transcriptomics

## Abstract

Cereal plants possess a fibrous root system in which crown roots form the major component. Crown roots develop post-embryonically from the lower, mostly underground nodes of the stem base. A strict spatiotemporal regulation of gene expression governs this process. Much of the knowledge about signaling pathways controlling crown root initiation (CRI) and development comes from rice. However, distinct regulatory mechanisms may have evolved in other cereals to adapt to different habitats.

In this study, using a Crown Root Inducible System (CRIS), we aimed to investigate the early molecular regulation of barley CRI. We revealed dynamic transcriptomic changes within the first 24 hours following auxin stimulation. Among the differentially expressed genes, we identified orthologs of important CRI regulators from other cereals, demonstrating that CRIS is suitable for uncovering genes involved in CRI. Further, ATAC-seq revealed that CRI relies on changes in chromatin accessibility near root development-related genes and within distal intergenic regions.

Finally, we focused on two transcription factors, HvNAC013 and CBF12C, which likely play roles in both CRI and abiotic stresses. By performing DAP-seq, we determined their genome-wide binding sites and identified their potential downstream targets. Data suggest that *CBF12C* is a putative target of HvNAC013, along with other auxin-responsive genes implicated in CRI. We propose that HvNAC013 and CBF12C function as part of a transcription factor network involved in CRI and potentially modulate root architecture in response to environmental conditions. This study enhances our understanding of the CRI mechanism in barley.

## Introduction

Barley (*Hordeum vulgare* L.), a cereal crop belonging to the *Triticeae* tribe, ranks fourth among cereals in global cultivation area (FAO 2024). In recent years, barley has become a model organism for cereal crop studies due to its advantageous characteristics, including self-pollination, diploid genome of moderate size and a low chromosome number (Von Bothmer et al. 2003). Advances in molecular tools, availability of mutant populations, large collections of landraces and wild accessions have fueled growing interest in functional genomics of barley (Russell et al. 2016, Szurman-Zubrzycka et al. 2018, Navrátilová et al. 2022). Barley’s adaptability to diverse environmental conditions has proven especially valuable for research on mechanisms of abiotic stress tolerance (Gürel et al. 2016).

Roots play a critical role in nutrient and water acquisition, anchorage, and plant-microbe interactions, making them a potential breeding target (Den Herder et al. 2010). One of the characteristic features of monocot plants, including cereals, is their fibrous root system. Although at the seedling stage the root system is composed of primary and seminal roots, later, the mature root system is essentially made of post-embryonic roots emerging from the stem, called crown roots (CR) (Hackett 1968). CR emerge from a subset of pericycle-like cells in the ground meristem of the stem base (Itoh et al. 2005, Coudert et al. 2013). Their initiation is primarily regulated by auxin and cytokinin that function antagonistically in the early stage of primordia establishment (Itoh et al. 2005, Zhao et al. 2009). The local accumulation of auxin serves as a positional signal for the emergence of a new root (Benková et al. 2003). The asymmetric auxin distribution is maintained by polar auxin transport, which directs auxin to specific cells in the ground meristem (Kitomi et al. 2008). The proper positioning of the auxin efflux carrier PIN-FORMED 1 (PIN1) is mediated by the rice (*Oryza sativa*) *CROWN ROOTLESS 4* (*CRL4*) gene, encoding a membrane-associated guanine-nucleotide exchange factor of the ADP-ribosylation factor G (GNOM) protein (Steinmann et al. 1999, Geldner et al. 2003, Kitomi et al. 2008). Auxin perception triggers a cascade of molecular events that lead to crown root initiation (CRI) (Inukai et al. 2005). Cytokinin signaling is involved both in initiation and in tissue differentiation during the development of CR primordia (Neogy et al. 2021, Omary et al. 2022).

The initiation of a new organ formation requires fine-tuning of gene expression. This process entails the binding of transcription factors to specific DNA sequences, located within regulatory regions, thereby activating or repressing transcription. The study of transcriptional regulators implicated in CRI initiated approximately two decades ago with the rice *crl1* mutant (Inukai et al. 2005, Liu et al. 2005). This mutant lacks CR in early development and the mature plants develop only a few CR. The *CRL1* gene encodes a transcription factor of the ASYMMETRIC LEAVES 2 (AS2)/LATERAL ORGAN BOUNDARIES (LOB) DOMAIN (LBD) protein family, and its expression is directly regulated by auxin through the AUXIN/INDOLE-3-ACETIC ACID(AUX/IAA)-AUXIN RESPONSE FACTOR (ARF) signaling pathway (Inukai et al. 2005; Liu et al. 2005). The observation that the adult *crl1* mutant plants still develop few CR suggests the existence of a CRL1-independent pathway (Inukai et al. 2005). Since these early studies, a complex network of signaling pathways regulating CRI, CR outgrowth and emergence was proposed in the rice model (Meng et al. 2019, Tanaka et al. 2023, Chennakesavulu et al. 2024).

Root development is regulated by hormones that respond to environmental stimuli, resulting in root plasticity (Zhou et al. 2021, Kang et al. 2022, Kim et al. 2022). Transcription factors are key to the transduction of this signal. The NO APICAL MERISTEM (NAM), ARABIDOPSIS TRANSCRIPTION ACTIVATION FACTOR (ATAF), and CUP-SHAPED COTYLEDON (CUC) (NAC) family acts in developmental, abiotic and biotic stress pathways (Han et al. 2023). Until now, 167 *NAC* genes have been identified in barley (Shen et al. 2009, Murozuka et al. 2018). However, only few of them have been functionally described (Kjaersgaard et al. 2011, Christiansen et al. 2016, Chen et al. 2023). The plant-specific transcription factor of the APETALA 2/ETHYLENE RESPONSIVE FACTOR (AP2/ERF) superfamily is involved in development and adaptive response to environment (Xie et al. 2019). In barley, AP2/ERF comprises 185 genes, divided into 3 families based on their domain composition (Sakuma et al. 2002, Ding et al. 2021). Tolerance to abiotic stresses is typically associated with members of the C-REPEAT BINDING FACTOR/DEHYDRATION RESPONSIVE ELEMENT BINDING 1 (CBF/DREB1) group (Stockinger et al. 1997, Yang et al. 2020). In rice, CRL1 regulates several NAC and AP2/ERF members, suggesting their involvement in CR formation (Coudert et al. 2015, Lavarenne et al. 2019). It appears that NAC and AP2/ERF transcription factors represent potential targets for improving both root architecture and abiotic stress tolerance in crops (Erpen et al. 2018).

The current study aimed to elucidate the genome-wide responses of barley seedlings during auxin-induced CR formation. We investigated the temporal transcriptomic changes within the first 24 h post-induction of CRI. We identified major transcriptional programs activated in the early phases of CRI and analyzed the involvement of chromatin accessibility in this process. Furthermore, we examined the possible role of HvNAC013 and CBF12C transcription factors in CR formation in barley. We identified potential targets of HvNAC013 and CBF12C, as well as the specific *cis*-regulatory elements recognized by these transcription factors. Based on our findings, we propose that HvNAC013 and CBF12C might function both in CR development and response to abiotic stress.

## Results and Discussion

### Crown root inducible system allows deciphering the dynamic transcriptomic changes controlling early crown root formation

The mature barley embryo contains two axillary meristem (AXM) primordia, one in the axil of the coleoptile and the other in the axil of the first leaf primordium; these primordia will later develop into tillers (Hussien et al. 2014). Primary and seminal root primordia appear from the basal pole of the embryo, and are protected by the coleorhiza (Jackson 1922, Hackett 1968, Aloni and Griffith 1991, Rossini et al. 2018). The first CR is formed within 3 days after germination (Nguyen et al. 2024), and 5 to 10 days after germination, the first CR emerge from the stem base (Hackett 1968, Knipfer and Fricke 2011).

**Figure 1:**
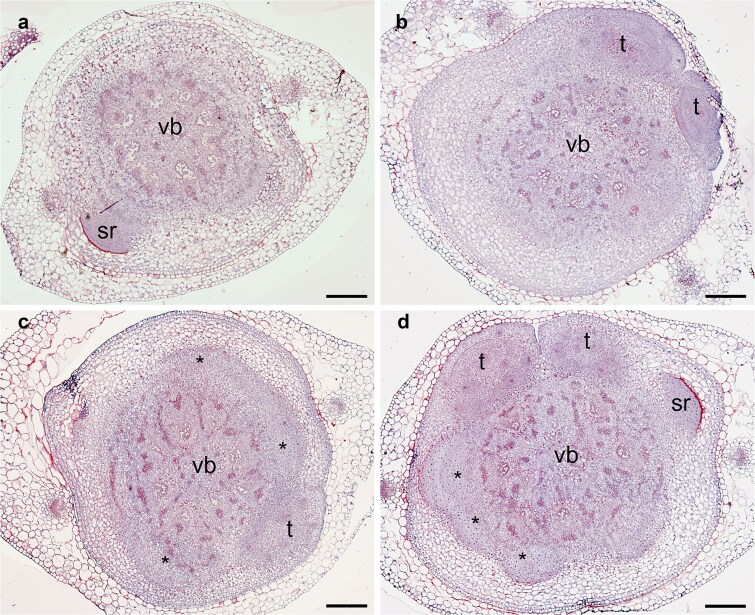
Cross-sections of stem base of barley seedlings grown in the crown root inducible system (CRIS). Seedlings were grown for 8 days in the presence of NPA, an inhibitor of polar auxin transport. Stem bases were collected before treatment with auxin (a) and at different times after auxin treatment: 24 h (b), 36 h (c), and 48 h (d). Cross sections (4 μm thick) were stained with periodic acid–Schiff and Naphthol blue black. Pictures were acquired with a Zeiss microscope with a 10x objective. Bar = 200 μm; asterisk stands for crown root primordium; sr, seminal root; t, tiller; vb, vascular bundles.

The unpredictable timing of CRI challenges a detailed molecular investigation. Therefore, to study the early events of CR primordium formation, we adapted the Lateral Root (LR) Inducible System (LRIS) that has been used in *Arabidopsis thaliana*, maize (*Zea mays*), *Medicago truncatula* and rice to study gene regulation of LR initiation (Jansen et al. 2013, Crombez et al. 2016, Motte et al. 2023). The Crown Root Inducible System (CRIS), similarly to LRIS, is based on the primary inhibition of CR by an inhibitor of the polar auxin transport (*N*-(1-naphthyl)phthalamic acid; NPA), followed by the induction by a synthetic auxin. In this system, the formation and emergence of seminal roots and tillers was not affected (Supplemental Fig. S1, S2), in agreement with previous reports (Woodward and Marshall 1988, Agusti and Greb 2013, Riaz et al. 2023). Barley seedlings did not develop any CR primordium when maintained in NPA for 8 days (Fig. 1a). The auxin treatment stimulated the synchronous initiation of few CR primordia (Fig. 1b-d). The CR primordia were observed 36 h after auxin induction (hai) (Fig. 1b, c) when they appeared as a mass of dividing cells. From 48 hai, we observed dome-shaped CR primordia (Fig. 1d). When seedlings were transferred from CRIS to large hydroponics with ½ Hoagland for three weeks, seedlings that were treated with auxin for 24 h developed significantly higher number of CR than the control plants (Supplemental Fig. S2). Further, our repeated histological studies revealed that auxin treatment consistently and repeatably induced the synchronous initiation of up to 6 CR primordia. Our results show that CRIS efficiently induced CR formation and suggest that CR priming and initiation occur within 36 hai. Therefore, we restricted our study to the first 24 h to identify the early molecular events of CRI.

**Figure 2:**
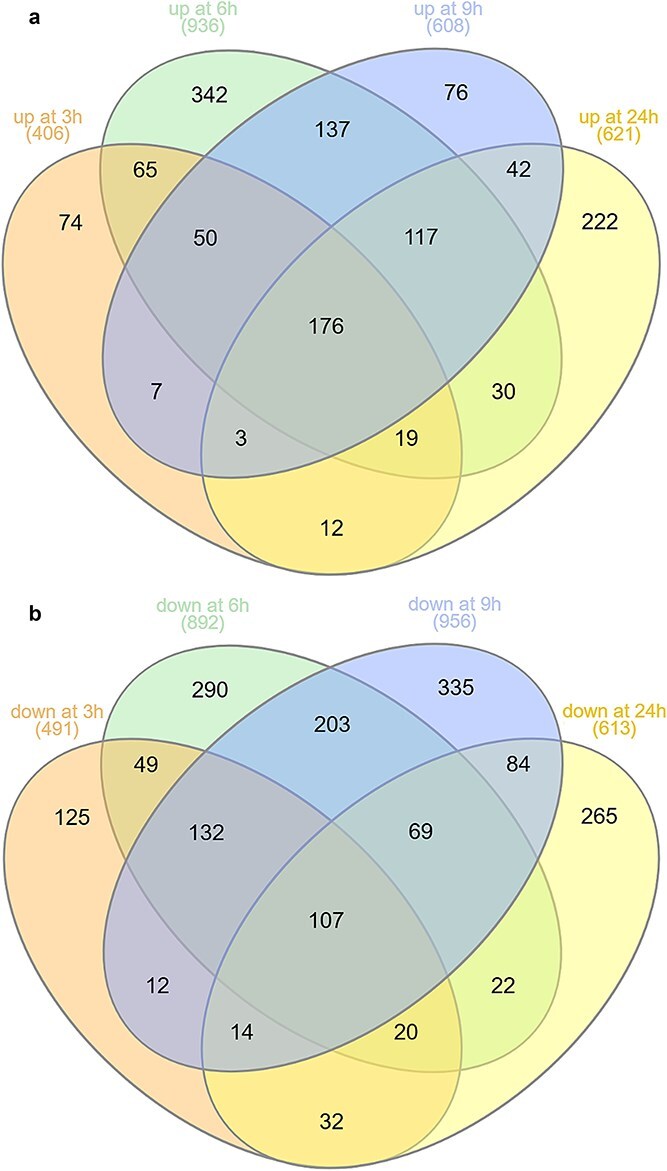
Venn diagrams showing overlap of up- (a) and down- (b) regulated genes in barley stem base at 3 h, 6 h, 9 h and 24 h after auxin treatment, (p_adj_ < 0.05). Diagrams were generated by InteractiVenn (Heberle et al. 2015).

We performed the whole transcriptome analysis of the stem base of barley seedlings collected before induction and at 3, 6, 9, and 24 hai (Supplementary Table S1A-S1D). Quality control parameters of the RNA-seq data are shown in Supplementary Fig. S3 and Supplementary Table S2A. Genes known to regulate the tiller number in barley and other cereals were not differentially expressed in our data, highlighting the suitability of the system to study genes related to CRI (Supplementary Table S1E). The number of significantly upregulated genes (p_adj_ < 0.05) ranged from 406 (3 hai) to 936 (6 hai) (Fig. 2a). The number of significantly downregulated genes (p_adj_ < 0.05) varied from 491 (3 hai) to 956 (9 hai) (Fig. 2b). We identified 176 and 107 genes, respectively up- and down-regulated across all time points (Supplementary Table S1F). The top genes exhibiting the highest activation included two *AUX*/*IAA* auxin signal repressors, *HvIAA28* and *HvIAA21*, not yet associated with CR development. Further, genes encoding proteins involved in phytohormone homeostasis were upregulated. These include two *GRETCHEN HAGEN 3* genes (*HvGH3–7* and *HvGH3–4*), which maintain biologically active auxin levels and are homologous to the *A. thaliana GH3.3* gene, known to be involved in adventitious rooting (Staswick et al. 2005, Gutierrez et al. 2012). One of the most induced genes encodes a gibberellin 2-beta-dioxygenase (*HvGA2ox6a*), suggesting that gibberellin deactivation is required for CRI in barley. This is supported by the observation that reduced gibberellin levels promote LR proliferation in poplar and that *GA2ox* is a downstream target of auxin signaling during CR formation in rice (Gou et al. 2010, Coudert et al. 2015). Genes encoding enzymes involved in the biosynthesis of cytokinins, ethylene and strigolactones were also induced across all time points. In contrast, a pronounced downregulation was observed for genes associated with JASMONATE ZIM-DOMAIN (JAZ) proteins and members of the AP2/ERF family (Supplementary Table S1F). JAZ proteins suppress jasmonate signaling and positively affect root growth (Li et al. 2017). To summarize, our data highlight an extensive hormonal crosstalk during auxin-mediated CRI in barley.

Genes associated with the auxin response were upregulated across the whole time series (Supplementary Figs. S4–S7, Supplementary Table S3). The initial 3 hai were characterized by an enrichment in transcriptional regulators for both the up- and down-regulated genes (Supplementary Fig. S4, Supplementary Table S3A). Biological processes such as ‘shoot and root development’, ‘regulation of hormone levels’ or ‘rhythmic processes’ were also enriched. In *A. thaliana,* the circadian clock regulates auxin signaling related genes and coordinates LR emergence (Voß et al. 2015). By 6 hai, genes related to ‘cellulose catabolism’ and ‘positive regulation of developmental growth’ were activated, while genes related to ‘cell wall organization and biogenesis’ were downregulated (Supplementary Fig. S5, Supplementary Table S3B). In *A. thaliana*, swelling of pericycle cells precedes the first division during LR initiation (Vermeer et al. 2014). Cell wall remodeling enzymes are likely required for loosening of the cell wall during this process, as well as for proper positioning of the asymmetric cell divisions (Lewis et al. 2013, Ramakrishna et al. 2019). At 9 hai, ‘acetyltransferases’ and ‘signaling receptor activators’ were specifically enriched, while ‘polyamine biosynthesis’ and ‘responses to abiotic stimuli’ were inhibited (Supplementary Fig. S6, Supplementary Table S3C). At 24 hai, we observed an enrichment in terms ‘cytokinin riboside 5´-monophosphate phosphoribohydrolase’ (cytokinin biosynthesis) and ‘negative regulation of cell population proliferation’ (Supplementary Fig. S7, Supplementary Table S3D). Conversely, cytokinin degradation and reactive oxygen species (ROS) metabolic processes were inhibited. The importance of ROS homeostasis for root architecture was recently described in rice, wherein a high number of ROS homeostasis-related genes were downregulated during CRI and CR outgrowth (Kumar et al. 2024).

### Auxin triggers fast upregulation of genes related to root apical meristem development

Recently, in barley, we identified *HvCRL1* as the ortholog of the rice *CRL1* and the maize *ROOTLESS CONCERNING CROWN AND SEMINAL ROOTS* (*RTCS*) gene that integrates both auxin and cytokinin to initiate CR formation (Hetz et al. 1996, Inukai et al. 2005, Liu et al. 2005, Taramino et al. 2007). In barley, using the CRIS, *HvCRL1* gene was significantly upregulated in the stem base of barley as soon as 1 h after auxin induction and *hvcrl1* knock-out harbored lower number of CR (Nguyen et al. 2025). We also characterized *HvCRL1L1* as the putative ortholog of the rice *DEGENERATED HULL 1* (*DH1*)/*OsLBD16* and the maize *LBD24* which functions in CR primordium initiation and outgrowth (Stelpflug et al. 2016, Garg et al. 2023, Geng et al. 2024). In our current dataset, both *HvCRL1* and *HvCRL1L1* transcripts strongly accumulated in response to auxin, with their expression remaining high during the whole duration of the experiment (Table 1, Supplementary Table S1). The transcript accumulation of these two genes, markers of CRI, confirms that the CRIS is suitable to uncover genes involved in the CRI in barley.

**Table 1:** List of barley orthologs of known crown root formation regulators from rice and their expression after auxin treatment

**Barley**		**Rice**		**LFC (p**_**adj**_ **< 0.05)**				**References**
**Gene name**	**Accession (HORVU.MOREX.r3.)**	**Gene name**	**Accession**	**3 h**	**6 h**	**9 h**	**24 h**	
*HvCRL1*	4HG0408280	*CRL1*	Os03g0149100	2.61	3.16	2.83	3.09	Inukai et al. 2005; Liu et al. 2005; Nguyen et al. 2025
*HvCRL1L1*	6HG0630410	*DH1/OsLBD16*	Os02g0820500	2.88	3.89	4.31	4.45	Garg et al. 2023; Geng et al. 2024; Nguyen et al. 2025
*HvQHB*	3HG0301330	*QHB*	Os01g0854500	1.84	2.68	2.38	2.70	Kamiya et al. 2003b; Coudert et al. 2015; Lavarenne et al. 2019
*HvCLE25*	6HG0632110	*OsCLE206*	Os02g0826300	-	1.35	1.85	1.89	Huangwei et al. 2013
*HvIAA13*	3HG0287760	*OsIAA6*	Os01g0741900	0.79	1.00	0.71	0.70	Lavarenne et al. 2019
*HvARF02*	7HG0726940	*OsARF19*	Os06g0702600	0.66	0.90	0.82	0.87	Lavarenne et al. 2019
*HvPIN1A*	6HG0615550	*PIN1A*	Os02g0743400	0.98	1.49	1.41	1.20	Lavarenne et al. 2019
*HvGH3–8*	3HG0294170	*OsGH3-1*	Os01g0785400	1.69	1.28	1.01	1.16	Lavarenne et al. 2019
*HvWOX11*	2HG0111270	*OsWOX11*	Os07g0684900	4.04	4.77	4.61	4.28	Zhao et al. 2009; Zhao et al. 2015; Geng et al. 2023, 2024
*HvCKX4*	3HG0318720	*OsCKX4*	Os01g0940000	2.61	3.03	3.20	3.24	Gao et al. 2014; Geng et al. 2023
*HvROP*	6HG0602690	*OsROP*	Os04g0561200	-	2.95	3.44	3.45	Gonin et al. 2022
*HvbHLH044*	4HG0406540	*OsbHLH044*	Os03g0188400	-	−0.74	−0.79	-	Lavarenne et al. 2019; Gonin et al. 2022
*HvERF3*	3HG0294880	*OsERF3*	Os01g0797600	-	0.77	0.75	0.42	Zhao et al. 2015
*RGF*	6HG0574910	*RGF*	Os02g0190700	-	1.78	2.61	2.19	Matsuzaki et al. 2010
*RGF*	2HG0205670	*RGF*	Os04g0643800	-	2.46	3.11	2.50	Matsuzaki et al. 2010
*HvPLT1*	2HG0203350	*OsPLT1*	Os04g0653600	-	-	0.65	-	Garg et al. 2022
*HvSCR*	2HG0168770	*OsSCR2*	Os12g0122000	1.21	1.30	1.65	-	Kamiya et al. 2003a

Our transcriptomic data revealed that auxin induced rapid changes in the expression of barley genes orthologous to genes controlling root initiation and development in rice (Table 1). The barley *QUIESCENT-CENTER SPECIFIC HOMEOBOX* (*HvQHB*) was induced within 3 hai and remained elevated thereafter. In rice, QHB is involved in the specification and maintenance of the root apical meristem stem cells (Kamiya et al. 2003b), suggesting that the root apical meristem stem cells specify fast after auxin induction.

In the first 3 hai, genes involved in auxin signaling, homeostasis and transport, such as *HvIAA13*, *HvARF02*, *HvPIN1A,* and *HvGH3–8*, were upregulated. HvIAA13 is a putative ortholog of the rice OsIAA6, a negative regulator of the CRL1-QHB interaction (Lavarenne et al. 2019). Concurrently, barley genes mediating auxin-cytokinin crosstalk were activated, including orthologs of the *WUSCHEL-RELATED HOMEOBOX 11* (*OsWOX11*), *RESPONSE REGULATOR 2* (*OsRR2*), and *CYTOKININ OXIDASE/DEHYDROGENASE 4* (*OsCKX4*). OsWOX11 integrates auxin and cytokinin signaling pathways and interacts with the CRL1 (Zhao et al. 2009, Geng et al. 2023, 2024). This interaction is required for the expression of *OsCKX4*, which controls cytokinin homeostasis during CRI and CR outgrowth (Gao et al. 2014, Geng et al. 2023). As cytokinin signaling maintains root meristem size by stimulating cell differentiation, during CRI, it must be repressed via cytokinin type-A RRs (Ioio et al. 2007, Kitomi et al. 2011).

Six hours after induction, orthologs of two other CRL1 targets were significantly differentially expressed: a *Rho GTPase* (*HvROP*) and a basic helix–loop–helix transcription factor (*HvbHLH044*). *HvROP* was upregulated, while the *HvbHLH044* was transiently downregulated. In rice, both genes promote crown root development (Coudert et al. 2015, Gonin et al. 2022). OsbHLH044 is a repressor of a gene network regulating programmed cell death and senescence during the later stages of root development (Gonin et al. 2022). Additionally, the *HvERF3* gene was upregulated. In rice, OsERF3 represses cytokinin signaling by positively regulating *OsRR2* during CR initiation, whereas it stimulates cytokinin signaling during CR emergence through its interaction with OsWOX11 to repress *OsRR2* (Zhao et al. 2015). A similar regulatory mechanism might operate in barley to control CR formation. Additionally, two genes from the *GOLVEN*/*root meristem growth factor* (*RGF*)/*CLAVATA3* (*CLV3*)/*ENDOSPERM SURROUNDING REGION* (*ESR*)-*related* (*CLE*)-*like* family were significantly upregulated. RGFs are essential for proper root growth, as they contribute to the root stem cell niche maintenance (Matsuzaki et al. 2010). *HvCLE25*, coding a CLE protein, was also found to be upregulated from 6 hai. In rice, the CLE/WOX module regulates root meristem maintenance and vascular tissue development (Huangwei et al. 2013).

By 9 hai, barley *PLETHORA 1* (*HvPLT1*) was significantly upregulated, in line with *OsPLT1* being expressed during rice CRI (Garg et al. 2022). OsPLT1 induces CR formation, likely through activating auxin biosynthesis genes (Garg et al. 2022). Our data reveal that *RGF* and *PLT* are sequentially expressed, consistent with described RGF-mediated control of PLT distribution patterns (Matsuzaki et al. 2010).

At 9 hai, the expression of *HvRR2* returns to its pre-induction state, and by 24 hai it is downregulated, diminishing cytokinin signaling repression. The repression of *HvRR2* might involve the ERF3/WOX11 complex, as described in rice (Zhao et al. 2015). Indeed, whereas *HvWOX11* is upregulated as soon as 3 hai, *HvERF3* expression increases from 6 hai, prior to the repression of *HvRR2*. Also, barley *SCARECROW* (*HvSCR*) gene, significantly upregulated between 3 and 9 hai, was no longer differentially expressed at 24 hai. In rice, *OsSCR* is specifically expressed in endodermal cells, where it is crucial for asymmetric cell divisions that generate cortex cell layers. Following division, *OsSCR* is downregulated in the daughter cells (Kamiya et al. 2003a). Our results suggest that in barley the cortex cell lineages are defined between 9 and 24 hai.

### Chromatin accessibility landscape specific for barley stem base was identified

In rice, a profound chromatin remodeling was suggested to occur in the CR primordium founder cells (Lavarenne et al. 2020, Garg et al. 2022). In barley, chromatin remodeling may be crucial during CRI as evidenced by the differential expression of genes encoding proteins involved in post-translational histone modification, nucleosome remodeling, chromatin structure and DNA homeostasis (Supplementary Table S1A–S1D).

**Figure 3:**
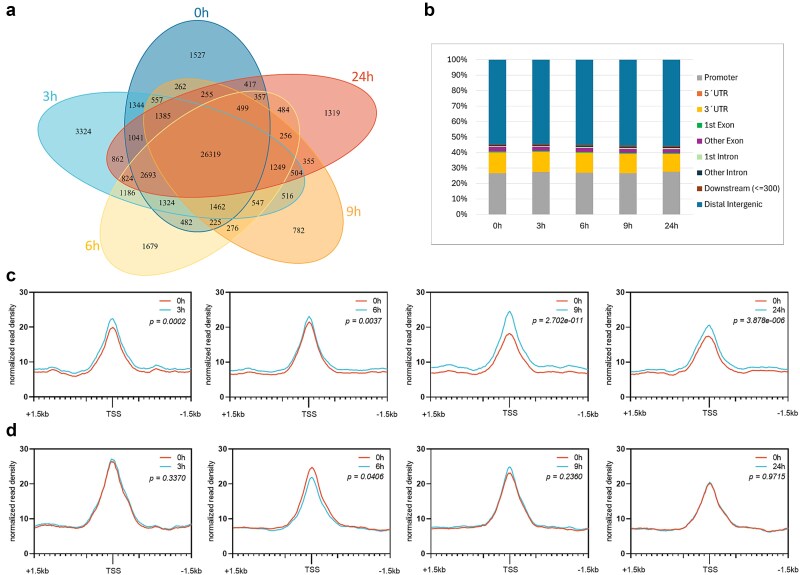
Comparison of ACRs identified in barley stem base at different times following auxin treatment and changes in ATAC-seq signal near the transcription start site (TSS) of differentially expressed genes. (a) Venn diagram illustrating the overlap of ACRs before induction (BI = 0 h) and the different time points (3 h; 6 h; 9 h; 24 h) following auxin treatment. (b) Distribution of ACRs relative to gene features, analyzed by ChIPseeker (Yu et al. 2015, Wang et al. 2022). (c, d) ATAC-seq read density ± 1.5 kb around TSS of significantly up- (c) or down-regulated (d) differentially expressed genes (p_adj_ < 0.05) after auxin treatment. Statistically significant if *P* < .01 (paired *t*-test).

Using the Assay for Transposase-Accessible Chromatin coupled to sequencing (ATAC-seq), we identified Accessible Chromatin Regions (ACRs) in barley stem base tissue primed for CR development (Buenrostro et al. 2013). Consistent with a high similarity among samples (Supplementary Fig. S8A), the number of ACRs remained relatively stable across all conditions, with 40 962 ATAC-seq peaks before auxin treatment (0 h), 45 760 peaks after 3 h of treatment, 40 145 peaks after 6 h, 36 397 peaks after 9 h, and 39 579 peaks after 24 h (Supplementary Table S2B). The comparative analysis revealed 26 319 peaks common to all time points (Fig. 3a). Only a minor proportion (up to 7.3%) of ACRs was located in unique genomic regions, likely reflecting the use of samples collected from identical tissue in a short time frame.

In ATAC-seq, the signal is typically enriched in active genomic regions around the transcription start sites (TSSs), in promoters and enhancers (Buenrostro et al. 2013). Here, we examined the spatial distribution of ACRs. The majority of ACRs were annotated to distal regions (Fig. 3b), with 60% of ATAC-seq peaks located within the 10 kb of a TSS, and half of these within 1 kb (Supplementary Fig. S8B-S8F), in agreement with the previous report (Lu et al. 2019). For our study, we considered a promoter as a region covering −500 bp upstream and + 100 bp downstream to the TSS (Pavlu et al. 2024). More than 25% of ACRs were assigned to promoter regions, while ~ 13% of them were associated with 3´UTRs (Fig. 3b).

TSS regions of upregulated genes displayed significantly higher ATAC-seq signals after auxin treatment (Fig. 3c). The highest increase in chromatin accessibility was observed at 9 h, coinciding with the enrichment of acetyltransferases (Supplementary Table S3C). In contrast, no significant change in chromatin accessibility around the TSS (*P* < .01) was observed for downregulated genes (Fig. 3d). Altogether, our data suggest that, in barley, chromatin remodeling contributes to the activation of genes regulating CR development, whereas transcriptional inactivation might rather involve transcriptional repressors, DNA methylation, or other non-coding elements (Zhang et al. 2006, Barrett et al. 2012).

### Changes in chromatin accessibility are important for controlling gene expression during CRI

**Figure 4:**
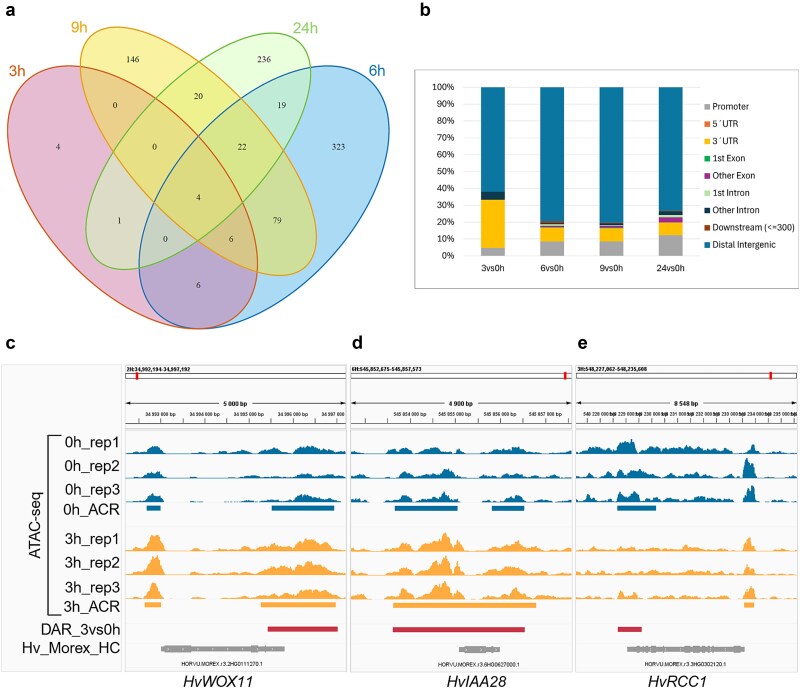
Comparison of differentially accessible regions (DARs) identified in barley stem base at different time points following auxin treatment and their distribution in the genome. (a) Venn diagram illustrating the overlap of DARs at different time points (3 h; 6 h; 9 h; 24 h) following auxin treatment. (b) Distribution of DARs relative to gene features, analyzed by ChIPseeker (Yu et al. 2015; Wang et al. 2022). (c-e) examples of DAR positions in the genome relative to selected differentially expressed genes potentially involved in CrI. ATAC-seq signal distribution at 0 h and 3 h after auxin treatment. Rectangles represent accessible chromatin regions (ACRs) at 0 h and 3 h, DARs, or genes. Genome visualizations in (c-e) were generated using the integrative genomics viewer (Robinson et al. 2011).

*Cis*-regulatory elements are key determinants of the tight spatiotemporal gene regulation that determines cell and tissue fate specification during plant development (Weber et al. 2016). Genomic sites that undergo significant changes in chromatin accessibility are termed Differentially Accessible Regions (DARs) and represent potentially essential *cis*-regulatory elements. Given the high similarity of ATAC-seq data across our experimental conditions, only a small proportion of ATAC-seq peaks were characterized as DARs (logCPM > −2, FDR < 0.15; Supplementary Tables S2C and S4). Only 21 DARs were recognized at 3 hai while a maximum of 459 sites were identified at 6 hai (Supplementary Tables S2C and S4). Most DARs were specific to their respective time points (Fig. 4a) and were located in distal intergenic regions (Fig. 4b). Distal ACRs are proposed to act as transcriptional enhancers controlling gene expression while interacting with their target genes via chromatin looping (Lu et al. 2019, Ricci et al. 2019).

We focused on DARs located within the genic and proximal regions, excluding ‘distal intergenic’ annotations. Between 17 to 50% of DARs were associated with DEGs (Supplementary Table S2C). A notable alteration in the chromatin accessibility was detected in the 3´-UTR region of *HvWOX11* as early as 3 hai (Fig. 4c). Later on, DARs were also observed in the promoter and distal upstream region of *HvWOX11* (Supplementary Table S4B). Recently, Geng et al. (2024) showed that OsWOX11 binds the histone demethylase JMJ706 to remove histone H3 lysine 9 (H3K9me2) mark from the *OsLBD16* promoter, enabling *OsLBD16* expression. Similarly, OsWOX11 recruits the ALTERATION/DEFICIENCY IN ACTIVATION 2/GENERAL CONTROL NON-DEPRESSIVE histone acetyltransferase complex to regulate genes involved in auxin transport, cell wall biosynthesis, and energy metabolism, all required for cell proliferation in the developing root meristem (Zhou et al. 2017). Altogether, it suggests that *HvWOX11* expression is regulated at the chromatin level. Assuming that the function of WOX11 is conserved among cereals, HvWOX11 might in turn recruit chromatin modifying enzymes to regulate downstream genes during CRI (Zhou et al. 2017, Geng et al. 2024).

The ATAC-seq signal spanning ~ 3.5 kb over *HvIAA28* at 3 hai (Fig. 4d) correlated with its expression pattern (Supplementary Table S1A). Later on, chromatin accessibility increased in the regulatory regions of other auxin-responsive genes, such as different *AUX*/*IAAs*, *HvGH3–4*, or F-box encoding genes (Supplementary Fig. S9A, Supplementary Table S4). Similar regulatory patterns were observed across many other genes including *LONELY GUY* (*LOG*), *HvGA2ox6a* (Supplementary Fig. S9B), a putative *JmjC domain-containing histone demethylase* (Supplementary Fig. S9C), and a gene encoding a member of the PUMILIO family of RNA-binding proteins, which is implicated in shoot stem cell maintenance and root cell proliferation (Supplementary Fig. S9D; Francischini and Quaggio 2009; Huang et al. 2014).

Early downregulation at the chromatin level was noted in the 3´-UTR region of the barley *REGULATOR OF CHROMOSOME CONDENSATION 1* (*HvRCC1*), correlating with its decreased expression (Fig. 4e). Later, downregulated DARs were identified near *EXORDIUM-like 1* (*HvEXL1*) (Supplementary Fig. S9E), or a gene encoding a cell wall restructuring enzyme, xyloglucan endotransglucosylase/hydrolase (XTH) (Supplementary Fig. S9F). Downregulated DARs spanned genomic regions enriched in transcription factor binding motifs, which thus became less accessible after auxin induction. Surprisingly, in two conditions (6 hai and 9 hai), the number of recognized transcription factor binding motifs was considerably higher in downregulated DARs compared to upregulated DARs (Supplementary Table S5). Additionally, DNA-binding motifs specific to certain transcription factor families, such as ERFs and AGAMOUS-LIKE, were more frequent in the downregulated DARs.

Compared to the transcriptomic changes during CRI, chromatin accessibility alterations appear less dynamic. This might reflect generally lower sensitivity of DARs detection, or suggests that the transcriptional regulation of the early stages of CRI relies on qualitative recruitment of key genomic sites, rather than widespread quantitative changes in chromatin accessibility.

### HvNAC013 and CBF12C show distinct expression patterns during CRI

To gain insights into the molecular cascade leading to CR formation, we focused on barley orthologs of the rice *OsNAC39* (Os03g0327100) and *OsERF28* (Os08g0545500), which are direct targets of CRL1 with a possible role in CR development. OsNAC39 is a transcription factor of the NAC family, which is part of the CRL1-dependent gene regulatory network (Coudert et al. 2015, Lavarenne et al. 2019). Beyond its role in CR formation, it is upregulated under drought and salinity stresses and by abscisic acid (Jeong et al. 2010, Lima et al. 2015, Ahn et al. 2017, Neogy et al. 2019). *OsERF28* is upregulated in a CRL1-dependent manner during the early stage of CR formation, but also in roots in response to drought, acetic acid, jasmonate and abscisic acid, suggesting a role in drought avoidance through root plasticity (Oh et al. 2009, Lavarenne et al. 2019, Ogawa et al. 2021).

**Figure 5:**
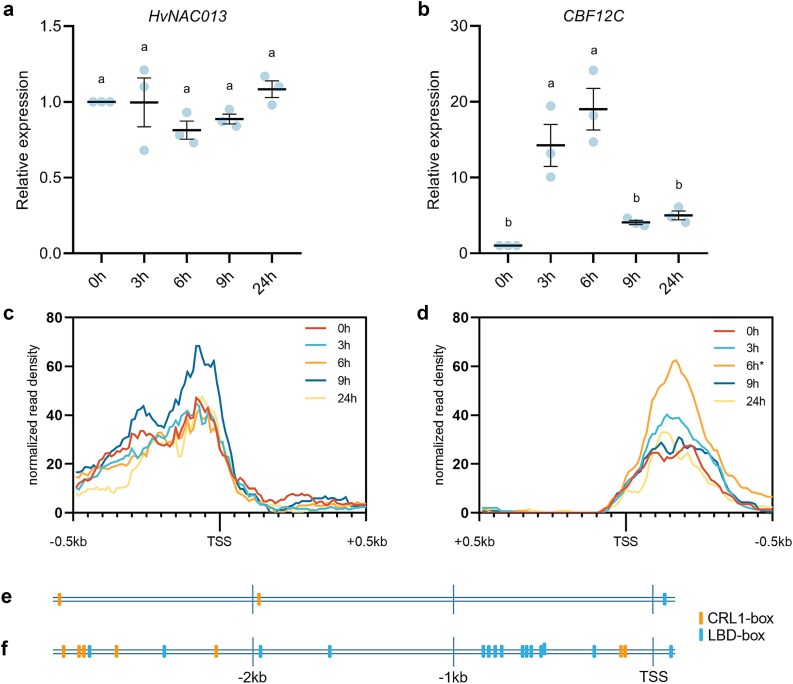
Gene expression of *HvNAC013* and *CBF12C* and the position and accessibility of *cis*-regulatory elements in their promoters. (a, b) gene expression of *HvNAC013* (a) and *CBF12C* (b) in barley stem base at different time points after auxin treatment, relative to the stem base before auxin treatment (0 h). Presented data show the mean ± standard error of the mean of three biological replicates. The lowercase letters indicate statistically significant differences between groups (one-way ANOVA followed by Tukey’s multiple comparison of mean rank, considered significantly different if p_adj_ < 0.05, analyzed in GraphPad prism 8.0.1). Groups sharing the same letter are not significantly different. (c, d) ATAC-seq read density ± 0.5 kb around transcription start site (TSS) of *HvNAC013* (c) and *CBF12C* (d). TSS regions are shown in opposite orientations, as the genes lie on different strands. Differentially accessible regions at 6 hai is marked by *. (e, f) position of LBD-box (GCGGCG) and CRL1-box (CACA[a/C]C) 3 kb upstream to 100 bp downstream of *HvNAC013* TSS (e) and *CBF12C* TSS (f).

In line with previous phylogenetic analyses, we identified *HvNAC013* as the ortholog of *OsNAC39* (Supplementary Fig. S10; Christiansen et al. 2011; Murozuka et al. 2018). The role of *HvNAC013* in CR development was recently suggested in barley seedlings grown in normal condition (Nguyen et al. 2024). Our data indicate that *HvNAC013* is steadily expressed across all time points following auxin treatment, which was validated by qPCR (Fig. 5a). Chromatin accessibility in its promoter region did not change significantly over the time (Fig. 5c). Consistent with the auxin-independent expression pattern of *HvNAC013*, auxin response elements were absent in the −3 kb and + 100 bp region of TSS. Instead, we identified *cis*-elements responsive to other phytohormones, light or low temperature (Supplementary Table S6A). Furthermore, the TSS region of *HvNAC013* was screened for the typical LBD binding motif, LBD-box (GCGGCG), and for the recently described CRL1-box (CACA[A/C]C) (Husbands et al. 2007, Gonin et al. 2022). An LBD-box was identified 43 bp downstream of a start codon, and the CRL1-box was represented twice (2 kb and 3 kb from TSS) (Fig. 5e). It is unlikely that *HvNAC013* is a direct target of HvCRL1. Indeed, LBD transcription factors function as homodimers or heterodimers, preferentially binding two DNA motifs separated by a spacer sequence, following mechanism identical to the molecular caliper described for ARF transcription factors (Korasick et al. 2015, Lee et al. 2017, Chen et al. 2019).

Concerning *OsERF28*, 10 potential barley orthologs were identified (Supplementary Fig. S11). *OsERF28*, also called *OsDREB1I*, is a member of the CBF3 subgroup of the CBF/DREB1 subfamily (Skinner et al. 2005, Moon et al. 2019). Therefore, we narrowed down the selection to 7 barley genes belonging to the same phylogenetic group (Skinner et al. 2005, Pasquariello et al. 2014). *CBF12C* was the only member of the CBF3 subgroup that was upregulated after auxin treatment (Supplementary Table S7). In contrast to *HvNAC013*, both transcriptomic and qPCR analyses showed that the expression of *CBF12C* was induced as early as 3 hai, reaching a maximum at 6 hai (Fig. 5b, Supplementary Table S1A–S1D). Chromatin accessibility in the promoter region of *CBF12C* increased transiently between 3 and 6 hai and this region was recognized as a significant DAR (Fig. 5d, Supplementary Table S4B). We identified two auxin responsive elements in the ~ 2.5 kb upstream region of the TSS (Supplementary Table S6B). Other *cis*-regulatory elements were associated with response to drought, light, methyl jasmonate, abscisic acid, or salicylic acid. In addition, the analysis revealed four dehydration-responsive elements (DREs), involved in responses to low temperature and water deficit, which are recognized by AP2/ERF transcription factors (Stockinger et al. 1997, Liu et al. 1998). Additionally, we discovered several LBD-box and CRL1-box elements in the −3 kb and + 100 bp region of TSS (Fig. 5f). The spacing of these motifs would likely allow dimerization of LBDs, as some are separated by only between 3 and 26 bp (Chen et al. 2019). In brief, our data suggest that *CBF12C* might be (i) a direct target of CRL1 and (ii) regulated by a transcription factor of the AP2/ERF family.

### HvNAC013 might regulate CRI through multiple downstream targets

In barley, the role of HvNAC013 in development, notably CR formation, is poorly understood. Therefore, we expressed HvNAC013 fused to a glutathione-*S*-transferase in bacteria. Using DAP-seq, we identified 95 779 and 77 690 HvNAC013 binding sites at 0 and 3 hai, respectively (Supplementary Table S2D). HvNAC013 binding sites were annotated to genes related to transcriptional regulation, cellular metabolic processes, organic substance transport, protein import into the nucleus, and hormone and stress responses (Supplementary Fig. S12, Supplementary Table S8A). Distal intergenic regions beyond ±3 kb from the TSS were excluded, thereby reducing the number of ‘3 hai’ peaks to 8466. Of these, 238 peaks were associated with genes differentially expressed at 3 hai (Supplementary Table S9). GO term enrichment analysis of upregulated genes reflected the studied conditions, being enriched in functions related to auxin response and cell communication (Supplementary Fig. S12, Supplementary Table S8B). Downregulated genes were enriched for transcription factors and genes involved in responses to heat and oxidative stress (Supplementary Fig. S12, Supplementary Table S8C).

**Figure 6:**
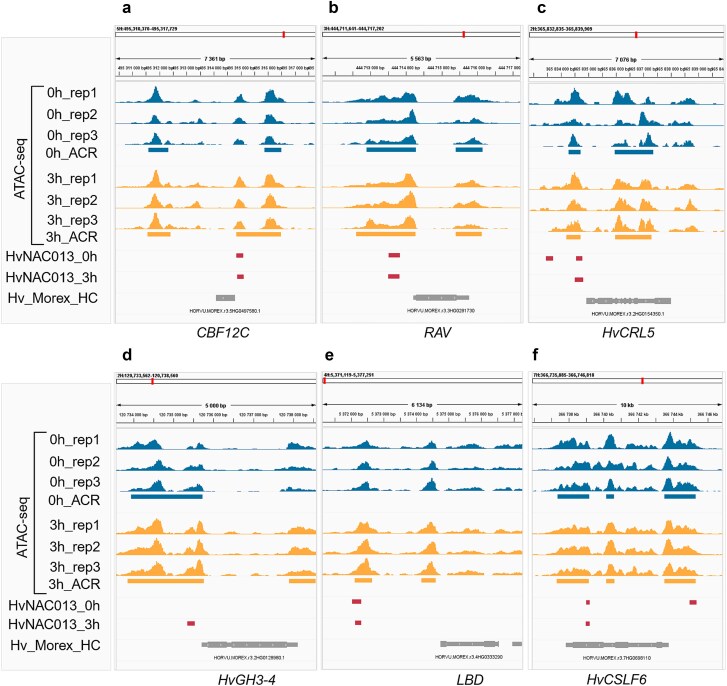
Examples of HvNAC013 binding sites near genes potentially involved in CRI. (a-f) position of HvNAC013 binding sites relative to selected genes and accessible chromatin regions (ACRs). ATAC-seq signal distribution is shown for 0 h and 3 h after auxin treatment. Rectangles represent ACRs at 0 h and 3 h, HvNAC013 DAP-seq peaks either at 0 h or 3 h, or genes. Genome visualizations were generated using the integrative genomics viewer (Robinson et al. 2011).

Interestingly, *CBF12C* appeared among highly upregulated genes whose promoter contains a HvNAC013 binding site (Fig. 6a). Transient accessibility of this site would allow binding of HvNAC013 upon auxin treatment, possibly contributing to the activation of *CBF12C* (Fig. 5d). Conversely, a HvNAC013 binding site in the promoter region of a *RAV* gene (belonging to the AP2/ERF) was associated with the downregulation of this gene (Fig. 6b). Another HvNAC013-binding site was identified in an ACR spanning the promoter of the putative barley *HvCRL5* (Fig. 6c), suggesting that *HvCRL5* may be regulated by HvNAC013. CRL5 is an auxin-induced member of the AP2/ERF family that promotes CRI in rice by repressing cytokinin signaling through positive regulation of type A-RR (Kitomi et al. 2011). The identification of *AP2*/*ERF* family members among HvNAC013 targets is consistent with NACs and AP2/ERFs functioning in the same signaling pathways (Chung et al. 2018, Dasgupta et al. 2020).

HvNAC013 belongs to the NAC-d subfamily, which includes regulators of LR initiation and middle cortex formation, as well as regulators of auxin and cytokinin biosynthesis and signaling pathways (Xie et al. 2000, Shen et al. 2009, Murozuka et al. 2018, Dong et al. 2019, Mao et al. 2020, Xie et al. 2023). We revealed that putative downstream targets of HvNAC013 include genes involved in auxin homeostasis and signaling, potentially associated with root development. Indeed, HvNAC013 binding sites were identified in the promoter of the auxin-responsive *HvGH3–4* gene (Fig. 6d) and in the 3´-UTR of *HvGH3–7*, both showing upregulation across all CRIS time points. Other binding sites were present within an intron of *HvARF02*, and in the distal region upstream of an *LBD* gene (Fig. 6e), ortholog of the *Arabidopsis LBD4*, involved in vascular proliferation (Smit et al. 2020).

Several NAC transcription factors possess both transcriptional repression and activation domains. The specific function they adopt is likely influenced by the cellular context and the presence of interacting partners (Xie et al. 2000, Hao et al. 2010, Mathew et al. 2016, Xie et al. 2023). In our data, about half of the HvNAC013 binding events were associated with significantly downregulated genes (Supplementary Table S9). One such gene encodes CELLULOSE SYNTHASE-LIKE F6 (HvCSLF6) (Fig. 6f), a key enzyme of mixed linkage glucan biosynthesis (Burton et al. 2006, Taketa et al. 2012). Interestingly, in rice, *OsCSLF6* is a direct target of OsWOX11 during CRI (Zhou et al. 2017).

DNA methylation is an important mechanism regulating gene expression by controlling the binding of activators and repressors (Zhang et al. 2006, Lang et al. 2017). In DAP-seq, DNA methylation can influence DNA-protein interactions (Bartlett et al. 2017). The analysis of Differentially Bound Sites (DBSs) allows for the identification of genomic sites exhibiting significant changes in transcription factor binding between two experimental conditions. In our study, binding sites identified at 3 hai were compared to the control (0 hai), yielding only 15 DBSs (MAnorm2, *P* < .01) linked to DEGs (Supplementary Table S9). This suggests that DNA methylation may not influence HvNAC013 binding under our experimental conditions.

Here, we propose several HvNAC013 target genes with a potential role in CRI. Considering the steady *HvNAC013* expression upon auxin treatment, the binding accessibility of *cis*-acting elements might be a crucial mechanism for regulation of its target genes. However, the vast number of other potential binding sites of HvNAC013 in the barley genome implies more complex roles of HvNAC013, likely targeting different sets of genes under various conditions.

### CBF12C might enhance expression of crown root development regulators in response to auxin

**Figure 7:**
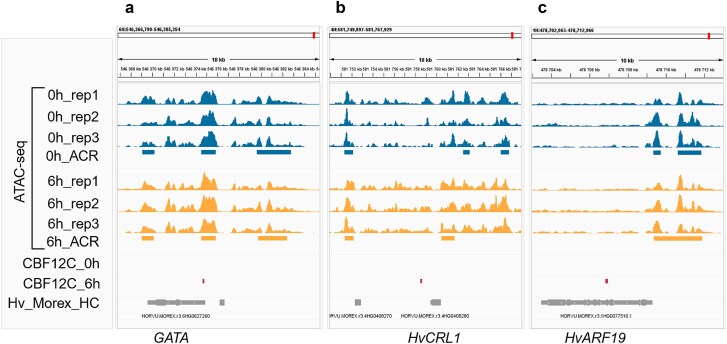
Examples of CBF12C binding sites near genes potentially involved in CRI. (a-c) position of CBF12C binding sites relative to selected genes and accessible chromatin regions (ACRs). ATAC-seq signal distribution is shown for 0 h and 6 h after auxin treatment. Rectangles represent ACRs at 0 h and 6 h after auxin treatment, CBF12C DAP-seq peaks either at 0 h or 6 h, or genes. Genome visualizations were generated using the integrative genomics viewer (Robinson et al. 2011).

We utilized DAP-seq to study genome-wide binding sites of CBF12C at 6 hai, when its highest expression was observed. The analysis generated 586 and 819 peaks at 0 and 6 hai, respectively (Supplementary Table S2D). Among the CBF12C binding sites associated with DEGs, only one was located in a promoter region, specifically −61 bp of the TSS of a *GATA* transcription factor (Fig. 7a, Supplementary Table S10), and overlapped with an ACR. Interestingly, *GATA* was differentially expressed only at 6 hai, when it was slightly downregulated. Another CBF12C binding site was associated with the *HvCRL1* gene (Fig. 7b). This site, positioned ~ 800 bp downstream of the *HvCRL1* gene, was part of a region enriched in ATAC-seq signal. Although the site of CBF12C binding was not specifically identified as an ACR, it could be part of a larger enhancer region. Additionally, a binding site was located in an exon of *HvARF19*, which displayed a substantially lower ATAC-seq signal compared to the adjacent areas (Fig. 7c). All 3 above-mentioned binding sites were recognized as DBSs (Manorm2, *P* < .01) in the 6 hai versus 0 hai comparison (Supplementary Table S10).

CBFs are mainly recognized for their role in enhancing cold tolerance in plants. However, growing evidence suggests that CBFs also contribute to root development. In *Arabidopsis*, CBF3 suppresses the growth of the primary root and LRs (Perez-Garcia et al. 2023). It is expressed in LR cell lineages that differentiate into endodermis/cortex or the quiescent center (Serrano-Ron et al. 2021). In the present study, we provide evidence that a member of the barley CBF family may function in CRI and auxin signaling by regulating downstream transcription factors, such as HvCRL1 and HvARF19.

### Both HvNAC013 and CBF12C bind DNA motifs associated with abiotic stress responses

**Figure 8:**
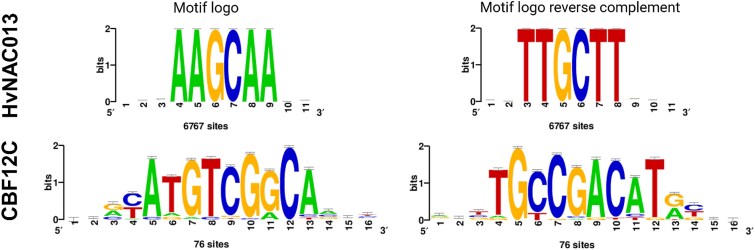
Best scoring HvNAC013 and CBF12C DNA-binding motifs. Identified from the set of DAP-seq peaks in the accessible chromatin regions. *De novo* motif identification was performed by RSAT plants *oligo analysis* tool (Thomas-Chollier et al. 2012).

To go deeper in our understanding of HvNAC013 and CBF12C function, we focused on their DNA binding motifs. In DAP-seq, transcription factors bind their preferred sequences without the constrains imposed by the chromatin state of the genomic region (Bartlett et al. 2017). Consequently, most of the DAP-seq peaks were found in distal intergenic regions, beyond 10 kb from TSS (Supplementary Fig. S13A, C). The co-analysis of ATAC-seq and DAP-seq data enabled the selection of transcription factor binding sites located within ACRs. This improves both the relative position to genes and the distance to TSS (Supplementary Fig. S13A–E). *De novo* motif identification was conducted in parallel on the complete set of DAP-seq peaks and on peaks within ACRs, revealing identical top-ranking motifs (Fig. 8, Supplementary Fig. S13F).

Members of the NAC transcription factor family can be divided into three clusters based on their DNA binding motifs: cluster 1 (T[G/A]CGT), cluster 2 (CCCGCC), and cluster 3 (TT[A/C/G]CTT) (Lindemose et al. 2014). Based on our analysis, HvNAC013 belongs to cluster 3, binding preferentially the TTGCTT motif (Fig. 8, Supplementary Fig. S13F, Supplementary Table S11A, S11B), and the TTACTT motif with a lower probability. These motifs were initially identified for the *Arabidopsis* ANAC056 and ANAC062 that are both involved in cold tolerance (Seo et al. 2010, Lindemose et al. 2014, Xu et al. 2024). Previously, HvNAC013 was used in electrophoretic mobility shift assay, where it bound to the CGT[G/A] NAC motif (Kjaersgaard et al. 2011). Here, motifs with CGT sequence were identified as well, however these were not bound preferentially by HvNAC013 in our assay (Supplementary Table S11).

*De novo* motif identification for CBF12C generated the best-scoring motif TGCCGACAT, with variations observed in the flanking sequences (Fig. 8, Supplementary Fig. S13F, Supplementary Table S11C, S11D). The identified motif contains the typical [G/A]CCGAC sequence of the C-repeat (CRT)/DRE, which is consistent with the binding patterns of other members of the barley CBF family (Skinner et al. 2005). The CRT/DRE *cis*-regulatory element is necessary for low temperature, drought and salt stress responses (Baker et al. 1994, Yamaguchi-Shinozaki and Shinozaki 1994, Stockinger et al. 1997). It was shown that CBF3 exhibits flexibility in binding the core motif with different flanking sequences and that the binding to the CRT/DRE motif is temperature-independent, unlike other CBFs (Skinner et al. 2005).

We identified transcription factor binding motifs for both HvNAC013 (TTGCTT) and CBF12C (TGCCGACAT) in the context of whole genomic DNA, extending our understanding of their transcriptional control during CRI.

### HvNAC013 and CBF12C are responsive to various abiotic stress conditions

When barley seedlings were subjected to water deficit (PEG) and salt stress, we observed a significant reduction in the number of CR (Supplemental Fig. S14A). Taking into consideration studies suggesting that *HvNAC013* and *CBF12C* are involved in abiotic stress responses (Kjaersgaard et al. 2011, Pasquariello et al. 2014), we examined the expression of *HvNAC013* and *CBF12C* in the shoot, stem base and roots of barley seedlings grown for 12 days under water deficit (PEG) and salt stress conditions.

Transcripts of *HvNAC013* accumulated in the stem base and to a higher extent in roots of seedlings grown under high salt concentration. Water deficit had no effect on its expression (Supplementary Fig. S14B). Publicly available datasets indicate an increase in *HvNAC013* expression in roots under salt stress and water deficit conditions. However, in studies where *HvNAC013* expression was tested over a time, its expression was either unchanged or lower at later time points (Klaus et al. 2024; PRJNA489775)*.*

Under control conditions, the expression of *CBF12C* is very low in all tissues analyzed. Salt stress induced a pronounced reduction in its expression in all tissues (Supplementary Fig. S14C). Further, water deficit strongly inhibited *CBF12C* expression in the shoot. Changes in the expression in stem base and roots were not significant. The inhibitory effect of abiotic stress on the expression of *CBF12C* was already reported in the shoot of barley plants subjected to drought for 4 days (Kokáš et al. 2016).

Our data demonstrate that *HvNAC013* and *CBF12C* are up- and down-regulated, respectively, in the stem base and roots in response to salt stress, suggesting that they may have a function in root system development in response to abiotic stresses.

The current study demonstrates that, while artificial, the CRIS offers the possibility to study CR initiation and development in barley. We generated an initial, unique transcriptomic resource that serves several important purposes, it is: (i) a valuable resource that can be cross-referenced with genes identified in CR development QTL studies, helping to prioritize candidate genes for functional characterization, (ii) a baseline for further studies that can examine these genes under various physiological conditions and developmental stages, and (iii) a foundation for meta-analyses with existing datasets from other species, enabling evolutionary and comparative studies of root development mechanisms across grasses and dicots.

We demonstrated that shortly after auxin induction, CRI is marked by an extensive phytohormone crosstalk and dynamic transcriptional changes, which facilitate proper expression of key regulators of root differentiation and development. Transcriptomic data combined to chromatin accessibility provided insights into the temporal gene regulation within the initial 24 h of CRI. Chromatin dynamics contributes to the recruitment of essential CRI regulators and influences the availability of *cis*-regulatory elements. We focused on members of the NAC and AP2/ERF families, whose role in CR formation has not been elucidated yet. During CRI, HvNAC013 could regulate several genes implicated in the auxin response, including *CBF12C*. We hypothesize that under non-stress conditions, HvNAC013 and CBF12C cooperate to stimulate CR formation and that they may have a different function in root development under certain abiotic stresses.

## Materials and Methods

### Plant material

For the whole study, the two-row spring barley (*H. vulgare* L.) cultivar Golden Promise was used.

### Crown root inducible system

The CRIS is adapted from the Lateral Root Inducible System (Crombez et al. 2016). Briefly, grains were poured in 70% ethanol for 30 s, rinsed with water, incubated for 10 min in a sterilization solution (4% sodium hypochlorite; 0.1% Tween-20), and finally extensively rinsed with sterile water. They were sown in Petri dishes on filter paper wet with 50 μM *N*-(1-naphthyl)phthalamic acid (NPA, Merck) in water. After 3 days at 4 °C in the dark, dishes were placed in a growth chamber (12 h/12 h; 13 °C night/16 °C day; light intensity: 170 μmol.m^−2^.s^−1^; 60% relative humidity). Seedlings with a 2 cm-long epicotyl were transferred for 4 days to hydroponics containing ½ Hoagland’s solution with 50 μM NPA. Thereafter, the solution was exchanged with ½ Hoagland’s solution supplemented with 50 μM 1-naphthaleneacetic acid (1-NAA, Merck). The stem base (2–3 mm fragment from the root-shoot junction) was excised with a scalpel and collected before treatment (control, 0 h), and at 3, 6, 9, and 24 hai. In this study, a single biological replicate was defined as a pool of the tissues (shoot, roots or stem base) of five seedlings grown in the same conditions. The pooling strategy was used to minimize the inherent variability of the individual samples, although it might affect the detection of very low-abundant transcripts (Taylor et al. 2019). For DAP-seq, two independent biological replicates were prepared, whereas three independent biological replicates were prepared for ATAC-seq and RNA-seq.

In parallel, plants that were grown according to the CRIS methodology were transferred to aerated hydroponics containing ½ strength Hoagland solution and grown for 3 weeks in phytochamber under controlled conditions (photoperiod: 12 h/12 h; 13 °C night/16 °C day; light intensity: 170 μmol.m^−2^.s^−1^; 60% relative humidity). After 3 weeks, the number of seminal roots, CR and tillers were determined.

### Stress treatment

For the stress experiment, grains were surface-sterilized and vernalized as described above, except that grains were sown on filter paper wet with sterile water. Five days after germination, barley seedlings were transferred to hydroponics with ½ Hoagland’s solution (control) or ½ Hoagland’s solution supplemented with 200 mM NaCl (saline stress) or 22% PEG_4000_ (water deficit). After 7 days, solutions were refreshed and plants were grown under control/stress conditions for another 5 days. The number of CR was manually determined. Further, shoots, stem bases and roots from 4 plants were collected for one biological replicate, immediately frozen in liquid nitrogen and stored at −80° until use. Experiments were repeated 7 times.

### Histological analysis

Barley stem bases were fixed overnight at 4 °C in a fixation buffer (4% [v/v] paraformaldehyde, 0.1 M phosphate buffer, pH 7). Samples were dehydrated through a graded EtOH series: 30, 50, 70, 90, 100% (v/v), each time for 30 min, and stored at 4 °C overnight. Samples were embedded in Technovit 7100 (Heraeus Kulzer) resin. Blocks were sectioned at 4 μm thickness using a HM 650 V microtome (Thermo Scientific). Slides were double-stained with Periodic acid–Schiff reagent (PAS) for the detection of carbohydrate compounds and Naphthol blue black (NBB) for the detection of proteins. Stained sections were observed under AXIO Scope A1 (Zeiss) microscope. Photographs were acquired using Axiocam 305 color camera.

### Preparation of RNA-seq libraries

RNA was extracted from stem base samples according to Wang et al. (2012). RNA was treated with Turbo Dnase I (Thermo Fisher Scientific) and precipitated with LiCl (Thermo Fisher Scientific). RNA Integrity Number (RIN) was determined by 2100 Agilent bioanalyzer. Libraries were prepared using NEBNext® Poly(A) mRNA Magnetic Isolation Module and NEBNext® Ultra II Directional RNA Library Prep Kit for Illumina® (New England Biolabs).

### Preparation of ATAC-seq libraries

Stem bases were fixed as follows: 10 min under vacuum in 0.5% methanol-free formaldehyde (Thermo Fisher Scientific) in PBS, 5 min under vacuum in 125 mM glycine in PBS, and 3 washes in cold PBS. Samples were homogenized in liquid nitrogen, transferred to LB01 buffer (Doležel et al. 1989) and filtered through 20 μm CellTricks filter (Sysmex). DAPI (3 μg/ml) was added prior to the sorting of 25 000 G1-phase nuclei into 200 μl PBS containing protease inhibitors using a FACS Aria II SORP flow cytometer (Becton Dickinson Immunocytometry Systems, San José, USA). Libraries were prepared using ATAC-Seq kit (Active Motif) according to manufacturer’s instructions with modifications. Tagmentation was stopped by adding de-crosslink buffer (100 mM Tris–HCl, 0.4% SDS, 2 mM EDTA, 0.4 M NaCl; v/v) containing 20 μg of proteinase K, followed by an overnight incubation at 65 °C with shaking (500 rpm). DNA was purified with Zymo ChIP Clean and Concentrate kit (Zymo Research). Libraries were PCR amplified with Q5 polymerase using Nextera™-Compatible Multiplex Primers (Active Motif), as followed: 72 °C/5 min, 98 °C/30 s, 14 cycles: 98 °C/10 s, 63 °C/30 s, 72 °C/1 min. PCR-amplified libraries were purified with 1.2 volumes of SPRI beads.

### Preparation of DAP-seq libraries

Stem base samples were collected before treatment (0 h), at 3 hai for HvNAC013 and 6 hai for CBF12C. Samples were homogenized in liquid nitrogen. Samples were incubated at 60 °C for 1 h in the extraction buffer containing 40 μg/ml proteinase K (Thermo Fisher Scientific). After adding phenol/chloroform/isoamylalcohol (25: 24: 1; Merck), the sample was transferred to a Phasemaker™ tube (Thermo Fisher Scientific), incubated for 10 min and centrifuged at 14000 x *g* for 5 min. The aqueous phase was transferred to a new tube. Genomic DNA was isolated as described by Pallotta et al. (2000). Dry pellet was resuspended in 400 μl of TE buffer. Genomic DNA was fragmented by Covaris ultrasonicator for 180 s to reach 200 bp average target size. DNA end-repair, A-tailing and adaptor ligation were performed according to Bartlett et al. (2017), using NEBNext Adaptor (New England Biolabs). The quality control of adaptor-ligated libraries was performed by qPCR using gbSG PCR master mix (Generi Biotech) and NEBNext Multiplex Oligos for Illumina (96 Unique Dual Index Primer Pairs, New England Biolabs).

Coding sequences of HvNAC013 (HORVU.MOREX.r3.4HG0379700) and CBF12C (HORVU.MOREX.r3.5HG0497680) with a C-terminal glutathione-*S*-transferase (GST) tag were codon-optimized, synthetized by GenScript and introduced into the vector pET-28a(+) in the Rosetta 2 (DE3) pLysS (Merck) *Escherichia coli* strain. Five hundred ml of LB medium supplemented with 50 mg/L kanamycin and 35 mg/L chloramphenicol were inoculated with 5 ml of an overnight culture and incubated at 37 °C for 3–4 h (OD_600_ > 1) while shaking at 180 rpm. Culture was induced by 0.4 mM isopropyl β-D-1-thiogalactopyranoside and incubated overnight at 18 °C under shaking. Cells were harvested by centrifugation at 5000 x *g* for 20 min at 4 °C. The cell pellet was subsequently resuspended in HEPES buffer (pH 7.4) supplemented with Pierce™ Protease Inhibitor tablet (Thermo Fisher Scientific) and Dnase I (0.1 mg/ml, Roche), and incubated on ice for 30 min. Cells were lysed by ultrasonic homogenizer 3000 MP (BioLogics). The lysate was then centrifuged at 14000 x *g* for 30 minutes at 4 °C. Recombinant proteins were purified using Pierce™ Glutathione Agarose (Thermo Fisher Scientific). Proteins were stored in 50 mM Tris–HCl at pH 8.0, 50 mM NaCl, and 5% glycerol.

Protein-DNA co-incubations were performed as described in Bartlett et al. (2017) with minor modifications. Five to 10 μg of purified HvNAC013-GST and CBF12C-GST in PBS (total volume 400 μl) were incubated with MagneGST beads (Promega) for 90 min at 4 °C, while slowly rotating. Five hundred ng of adaptor-ligated DNA library, prepared as described above, were diluted in PBS and co-incubated with HvNAC013-GST or CBF12C-GST bound to MagneGST beads for 90 min at 4 °C, rotating horizontally. Control reactions were performed with free GST and a no-protein sample.

Eluted DNA was PCR enriched using Phusion DNA polymerase (New England Biolabs) and NEBNext Multiplex Oligos for Illumina (96 Unique Dual Index Primer Pairs, New England Biolabs) in the total volume of 50 μl. USER enzyme (New England Biolabs) was added into the PCR reaction and, prior to PCR, samples were incubated at 37 °C for 15 min. PCR was performed as described in Bartlett et al. (2017). PCR-enriched DNA libraries were purified twice using 0.9x AmpureXP beads (Beckman Coulter), resuspended in TE buffer and stored at −20 °C.

### Sequencing

Before pooling, all libraries were quantified using Library Quant Kit (New England Biolabs). DAP-seq libraries were sequenced on a NovaSeq 6000 SP flow cell (Illumina) producing 2 x 150 bp reads; ATAC-seq and RNA-seq libraries were sequenced on a NovaSeq 6000 S1 flowcell yielding 2 x 150 bp reads.

### Differential gene expression analysis

The quality of reads generated by sequencing was checked using the FastQC software (Andrews 2010). Adaptors were trimmed using TrimGalore v0.6.10 (Krueger 2023). Trimmed reads were then aligned by HISAT2 software v2.2.1 to the reference genome *H. vulgare* subsp. *Vulgare* (MorexV3_pseudomolecules_assembly; GCA_904849725.1) using default parameters (Kim et al. 2019, Mascher et al. 2021). Mapped reads were assigned to the genes by featureCounts v2.0.3 (Liao et al. 2014). Analysis of differential gene expression was performed using the DESeq2 and/or DEVIS software (Love et al. 2014, Price et al. 2019). DEGs were functionally BIN-annotated by Mercator (Schwacke et al. 2019). Functional enrichment analyses of GO terms was performed by g:Profiler (Kolberg et al. 2023).

### ATAC-seq data analysis

ATAC-seq data were processed according to Reske et al. (2020) with minor modifications. Nextera adaptors were removed and reads were quality filtered using TrimGalore v0.6.10 (Krueger 2023). Reads were aligned to the Morex V3 genome by bowtie2 v2.3.5.1 (Langmead and Salzberg 2012, Langmead et al. 2019, Mascher et al. 2021). The final set of ATAC-seq peaks was generated by calling naïve overlapping peaks from 3 biological replicates using the naiveOverlapBroad.sh script (Reske et al. 2020).

For DARs identification, bam files were subsampled and processed by csaw (Lun and Smyth 2014, 2016) according to method *IV* described by Reske et al. (2020). Consensus peak sets were prepared as a union of all replicate peak sets for compared conditions. Low abundance peaks were filtered by logCPM (counts per million) > −2 and the threshold for filtering final DA regions was FDR (false discovery rate) < 0.15.

MEME suite program SEA (v5.5.5) was used with default parameters for enrichment analysis of transcription factor binding motifs in DAR regions (excluding DARs annotated to distal intergenic regions beyond ±3 kb from TSS) (Bailey and Grant 2021).

All identified ATAC-seq peaks were associated with its nearest gene by ChIPseeker (Yu et al. 2015, Wang et al. 2022), using the settings defined in Pavlu et al. (2024). Peaks in promoters (−500 bp to +100 bp from TSS), within gene features (exons, introns), 5’UTR, 3’UTR, or up to 300 bp downstream, were annotated.

### DAP-seq data analysis

First, adapter and quality trimming were performed with TrimGalore v0.6.10 (Krueger 2023). Soft-masking of genome repetitive sequences of barley Morex V3 genome assembly (Mascher et al. 2021) was performed by Red repeat detector (Girgis 2015). Paired-end read alignment was performed by bowtie2 v2.3.5.1 with default settings (Langmead and Salzberg 2012, Langmead et al. 2019). Aligned reads were filtered based on mapping quality score using Samtools view -F 4 -q 30 (Li et al. 2009). Peak calling was done by MACS2 callpeak v2.2.7.1, setting the effective genome size to 4.6e+9 and otherwise using default settings (Zhang et al. 2008). The greenscreen workflow was utilized to remove potential artifactual signals (Klasfeld and Roulé 2022). MACS2 peaks of corresponding biological replicates were used in Multiple Sample Peak Calling tool to generate a consensus peak set for each experimental condition, setting weak *p*-value threshold to -w 1e-4 and stringent threshold to -s 1e-8 (Jalili et al. 2015).

Differential binding analysis between conditions before and after auxin treatment was performed by Manorm2, using a cutoff *P* < .01 (Tu et al. 2021). Input tables for the DA were generated by Manorm2_utils (https://github.com/tushiqi/MAnorm2_utils/tree/master).

### *De novo* transcription factor binding motif identification

Genomic sequences corresponding to the identified DAP-seq peaks were searched for overrepresented DNA binding motifs with RSAT peak motifs, selecting the *oligo-analysis* algorithm and using the Markov background model (Van Helden et al. 1998, Thomas-Chollier et al. 2012). The best binding motif was selected as being the most significant among all identified motifs. Motifs with significance value ≤ 2 and motifs consisting of simple repeats were excluded. Similar motifs were merged by RSAT matrix-clustering tool (Castro-Mondragon et al. 2017). All motifs were searched for matches in footprintDB (Sebastian and Contreras-Moreira 2014).

### Gene expression analysis by qPCR

For all gene expression analyses, cDNA was synthesized from 1 μg of total RNA by LunaScript® RT SuperMix Kit (New England Biolabs). Concentrated cDNA (stress treatment) or 5 times diluted cDNA (CRIS) was used in a reaction containing Luna® Universal Probe qPCR Master Mix (New England Biolabs), 400 nM primers and 200 nM probe (Supplementary Table S12). qPCR was carried out on a QuantStudio™ 7 Pro Real-Time PCR System (Thermo Fisher Scientific) as follows: 95 °C/1 min, 40 cycles: 95 °C/15 s and 60 °C/30 s. Three reference genes, determined as being the most stable across conditions, were used for normalization. For CRIS, reference genes were: *Elongation factor2* (*EF2α*), *Actin* (*Act*) and *Emsy N-terminus domain-containing protein* (*ENT*). For stress treatment, reference genes were: *EF2α*, *Act* and *Cyclic nucleotide-binding domain-containing protein* (*CNG*). Changes in gene expression were determined using the efficiency corrected 2 − ΔΔCt method (Pfaffl 2001), followed by log10 transformation of normalized relative quantities, mean centering and autoscaling (Willems et al. 2008). Each sample was analyzed in thee technical and three biological replicates.

### Gene orthologs and gene names

Gene orthologs were identified either by Ensembl Plants or SHOOT (based on OrthoFinder v3) (Emms and Kelly 2022, Yates et al. 2022). Gene names of *ARFs* were used according to Tombuloglu (2019), *AUX/IAA* family according to Shi et al. (2020), *GH3* according to Kong et al. (2019). Supplementary Table S13 lists the barley gene abbreviations used in the text along with their corresponding accession numbers.

## Supplementary Material

pcp-2025-e-00050-File011_pcaf077

pcp-2025-e-00050-File012_pcaf077

pcp-2025-e-00050-File013_pcaf077

pcp-2025-e-00050-File014_pcaf077

pcp-2025-e-00050-File015_pcaf077

pcp-2025-e-00050-File016_pcaf077

pcp-2025-e-00050-File017_pcaf077

pcp-2025-e-00050-File018_pcaf077

pcp-2025-e-00050-File019_pcaf077

pcp-2025-e-00050-File020_pcaf077

pcp-2025-e-00050-File021_pcaf077

pcp-2025-e-00050-File022_pcaf077

pcp-2025-e-00050-File023_pcaf077

pcp-2025-e-00050-File024_pcaf077

## Data Availability

The raw data related to RNA-seq, ATAC-seq and DAP-seq have been deposited in the SRA database, under the BioProject accession number PRJNA1218950. The processed data files generated in this study have been deposited in Zenodo (DOI: 10.5281/zenodo.14825880).
