# Supplementary material for: Temporal regulation of gene expression during auxin-triggered crown root formation in barley: an integrated approach
Source: Plant Cell Physiol. 2025 Jul 13;66(9):1284–303. doi: 10.1093/pcp/pcaf077 (PMC12461856; doi:10.1093/pcp/pcaf077)
Supplement: pcp-2025-e-00050-File011_pcaf077 [file pcp-2025-e-00050-file011_pcaf077.pdf]

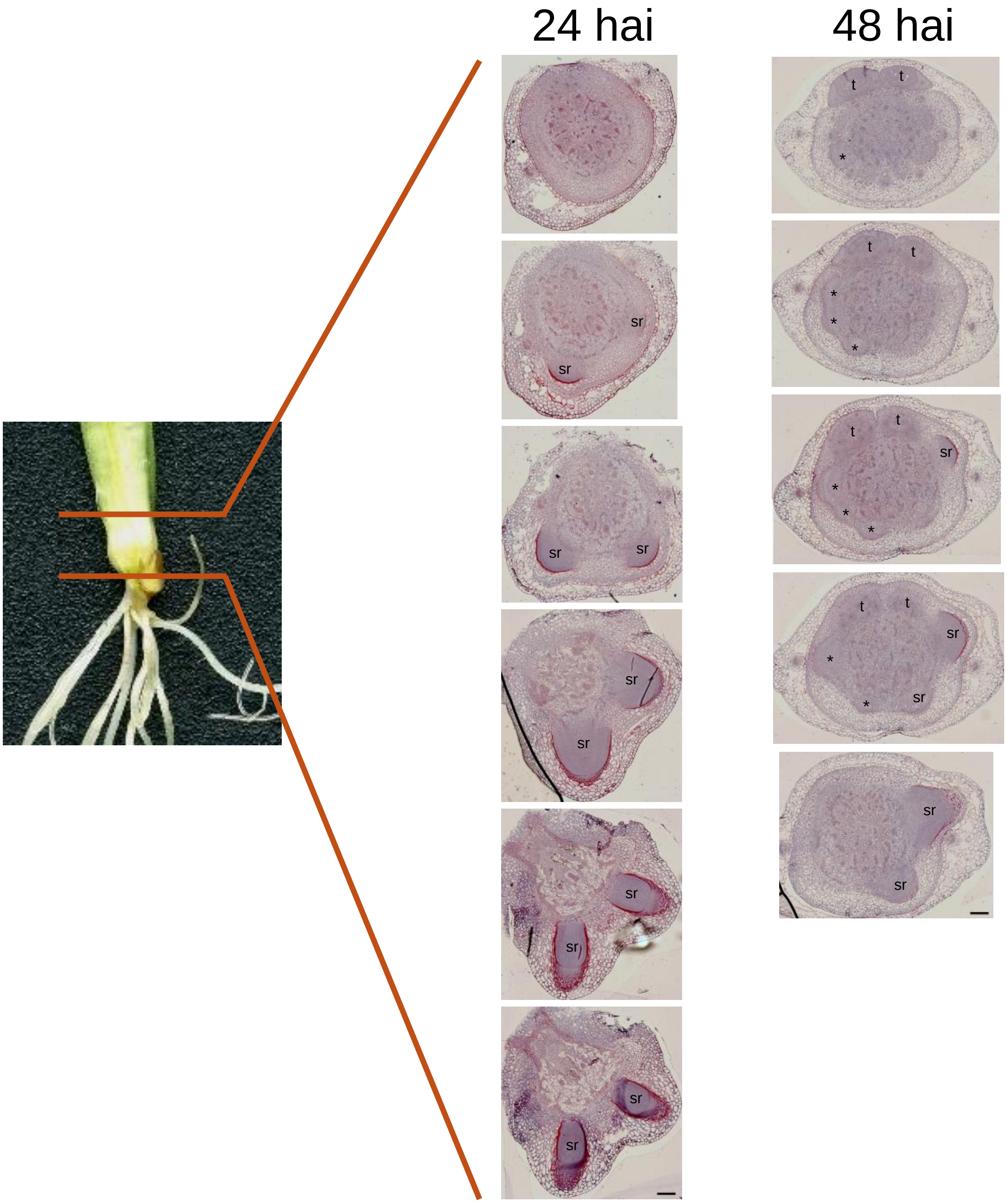

Figure S1. Serial cross-sections of stem base of barley seedlings grown in the Crown Root Inducible System (CRIS). After germination, seedlings were grown for 8 days in the presence of NPA, an inhibitor of polar auxin transport. Stem bases were collected at 24 h, or 48 h after 1-NAA treatment. Cross sections were 4 μm thick and stained with Periodic acid–Schiff and Naphthol blue black. Pictures were acquired with a Zeiss microscope with a 10x objective. Bar = 200 μm; asterisk = crown root primordium; sr, seminal root; t, tiller.

A

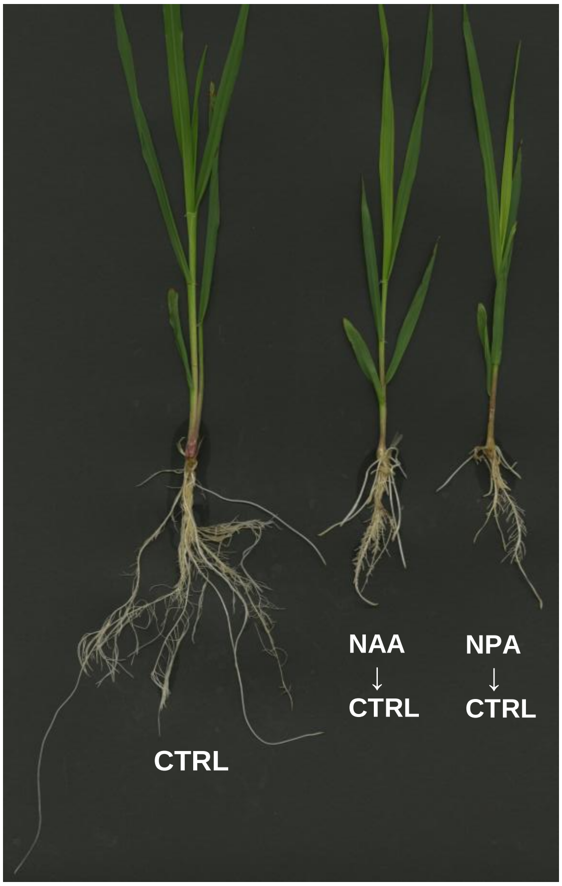

B

	CTRL (n=29)	NAA → CTRL (n=30)	NPA → CTRL (n=30)
Seminal roots	7.93 ± 0.15	7.43 ± 0.13	7.75 ± 0.13
Crown roots	5.90 ± 0.22	13. 97 ± 0.43****	7.80 ± 0.48
Tillers	0.28 ± 0.08	0.53 ± 0.10	0.40 ± 0.12

Figure S2. Effect of auxin treatment on the number of seminal and crown roots, and tillers. Barley seedlings were grown according to the Crown Root Inducible System (CRIS) methodology. After treatment, plants were grown for 3 more weeks in large hydroponics with aeration, the solution was changed once a week. Control plants were grown in ½ Hoagland for the whole duration of the experiment. A) From left: control (no treatment); 1-NAA treatment for 24 h, then transfer to ½ Hoagland; NPA treatment, then transfer to ½ Hoagland. B) Number of seminal and crown roots, and tillers. The numbers represent the mean ± standard error of the mean. Taking into consideration the Gaussian distribution of residuals and equals SD, statistical significance analyses were done either by a Brown-Forsythe and Welch ANOVA, or a non-parametric Kruskal-Wallis test (GraphPad Prism 10; **** $p < 0.0001$).

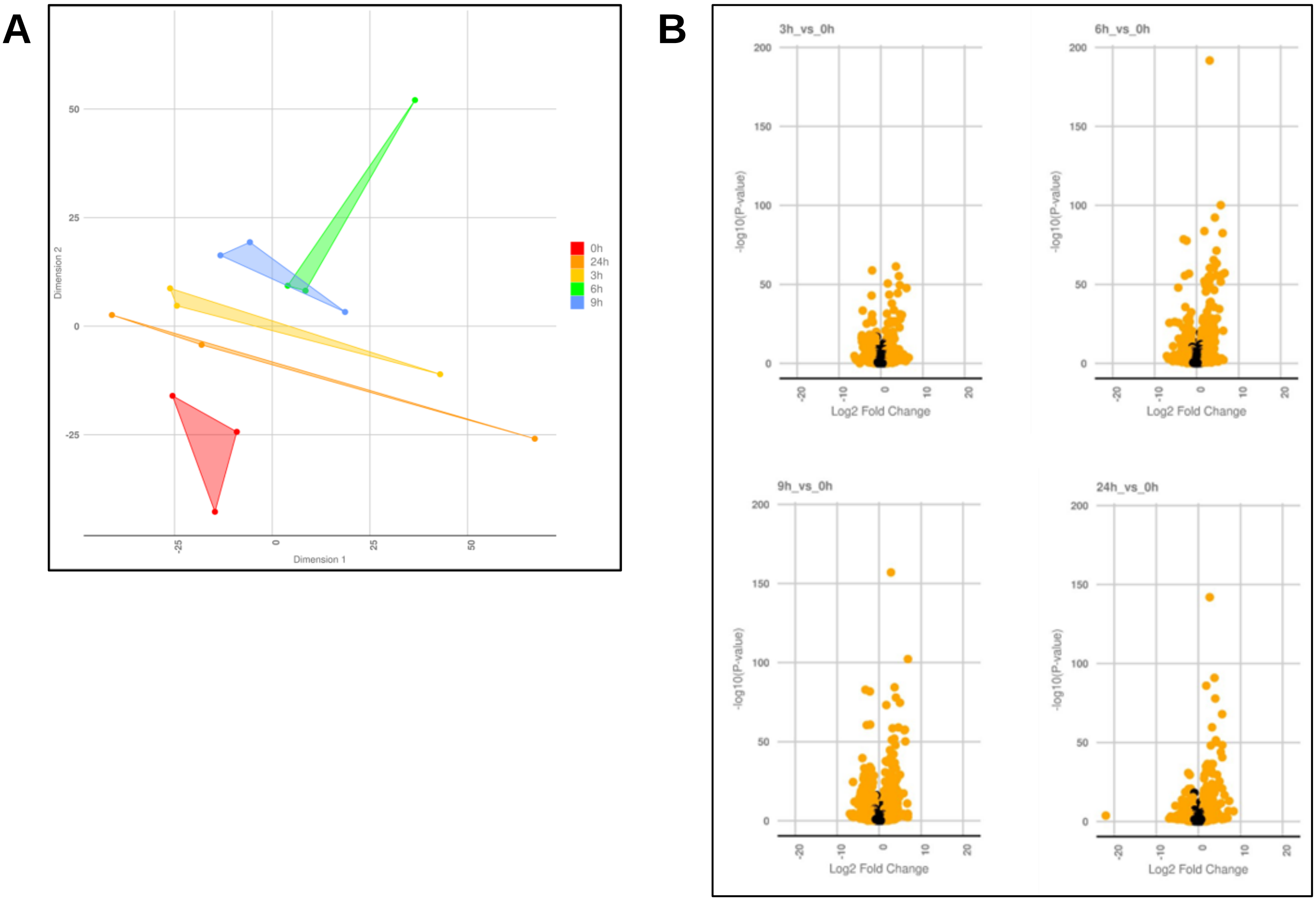

Figure S3. Quality control of RNA-seq data. (A) The multidimensional scaling hull plot of RNAseq data representing samples across four different time points, each with three replicates. Generated using the R package DEVis (Price et al. 2019). The plot reveals the relationships and similarities/dissimilarities between these samples. (B) Volcano plot of the log2 fold change and statistical significance (negative logarithm of the p-value) of expressed genes.

		GO description	GO
3h-UP	MF	DNA-binding transcription factor activity	GO:0003700
	BP	response to auxin	GO:0009733
		regulation of DNA-templated transcription	GO:0006355
		multicellular organism development	GO:0007275
		regulation of hormone levels	GO:0010817
		rhythmic process	GO:0048511
3h-DOWN	MF	DNA-binding transcription factor activity	GO:0003700
		sequence-specific DNA binding	GO:0043565
		phosphatase activity	GO:0016791
	BP	regulation of RNA metabolic process	GO:0051252
		response to abiotic stimulus	GO:0009628
		response to oxygen-containing compound	GO:1901700
		carotenoid biosynthetic process	GO:0016117

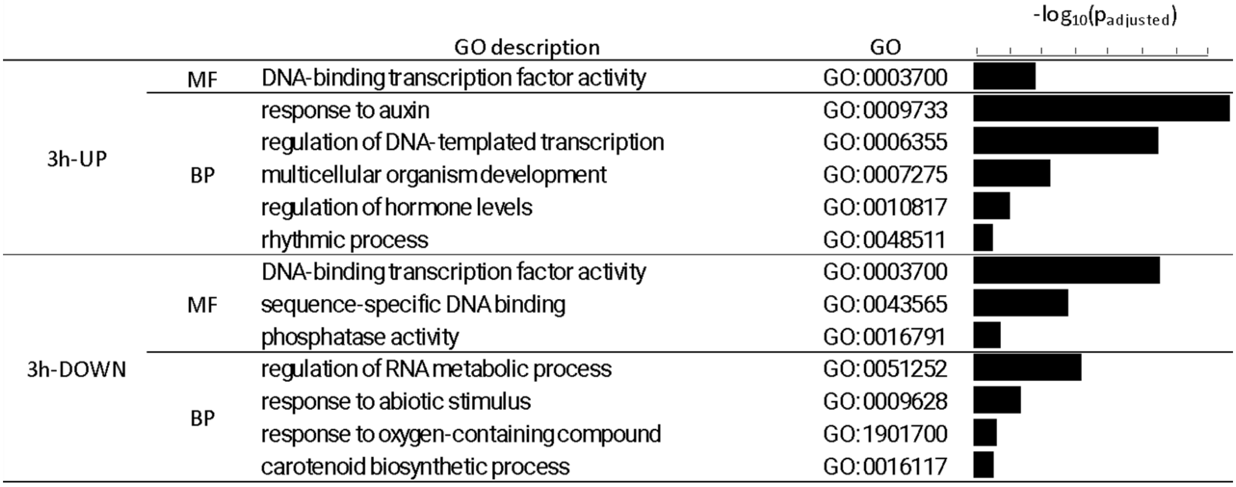

Figure S4. GO term enrichment for differentially expressed genes (DEGs) identified in the stem base of barley at 3 h following auxin treatment. Functional enrichment analyses of GO terms was performed by g:Profiler, using the feature for driver terms selection (Kolberg et al. 2023). 3h_UP, DEGs up-regulated at 3 h; 3h_DOWN, DEGs down-regulated at 3 h; MF, molecular function; BP, biological process.

		GO description	GO
6h_UP	MF	DNA-binding transcription factor activity	GO:0003700
		DNA binding	GO:0003677
		cellulase activity	GO:0008810
	BP	response to hormone	GO:0009725
		developmental process	GO:0032502
		adventitious root development	GO:0048830
		positive regulation of developmental growth	GO:0048639
		cellulose catabolic process	GO:0030245
		rhythmic process	GO:0048511
		regulation of hormone levels	GO:0010817
		hormone biosynthetic process	GO:0042446
6h-DOWN	MF	glycosyltransferase activity	GO:0016757
		DNA-binding transcription factor activity	GO:0003700
		sequence-specific DNA binding	GO:0043565
		hydrolase activity, hydrolyzing O-glycosyl compounds	GO:0004553
		RNA polymerase II cis-regulatory region sequence-speci	GO:0000978
		sucrose alpha-glucosidase activity	GO:0004575
	BP	carbohydrate metabolic process	GO:0005975
		cell wall organization or biogenesis	GO:0071554
		response to heat	GO:0009408

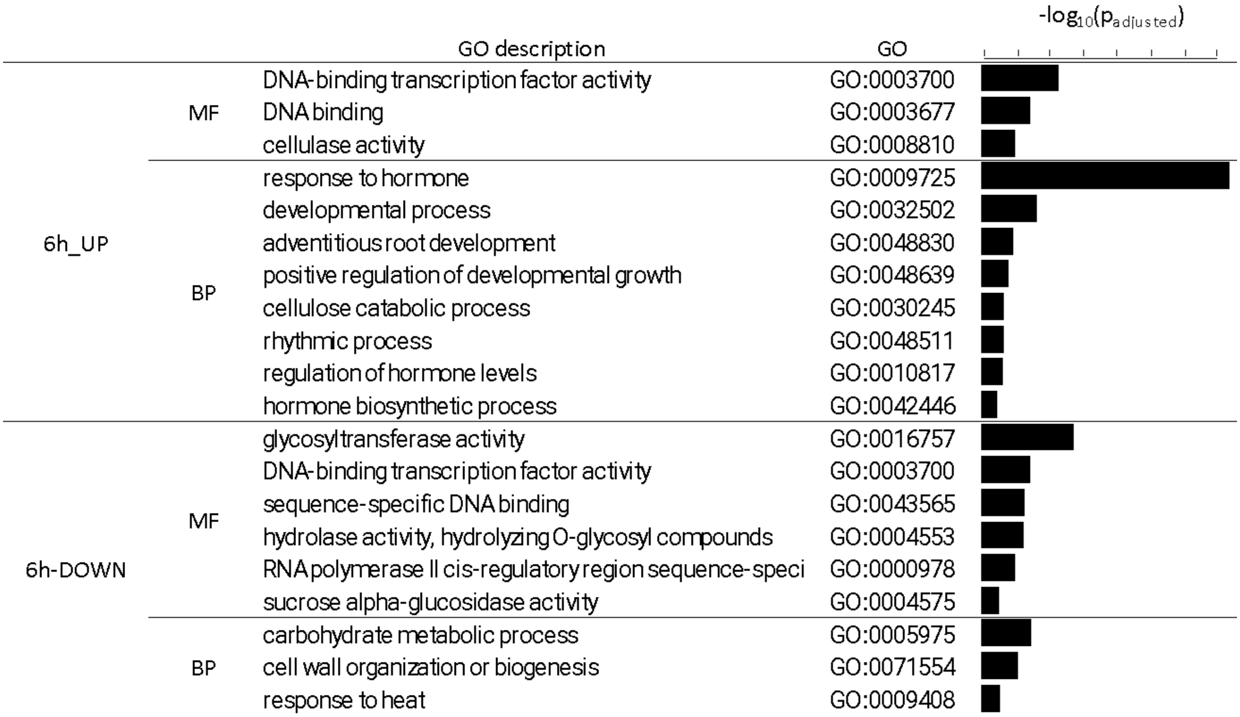

Figure S5. GO term enrichment for differentially expressed genes (DEGs) identified in the stem base of barley at 6 h following auxin treatment. Functional enrichment analyses of GO terms was performed by g:Profiler, using the feature for driver terms selection (Kolberg et al. 2023). 6h_UP, DEGs up-regulated at 6 h; 6h_DOWN, DEGs down-regulated at 6 h; MF, molecular function; BP, biological process.

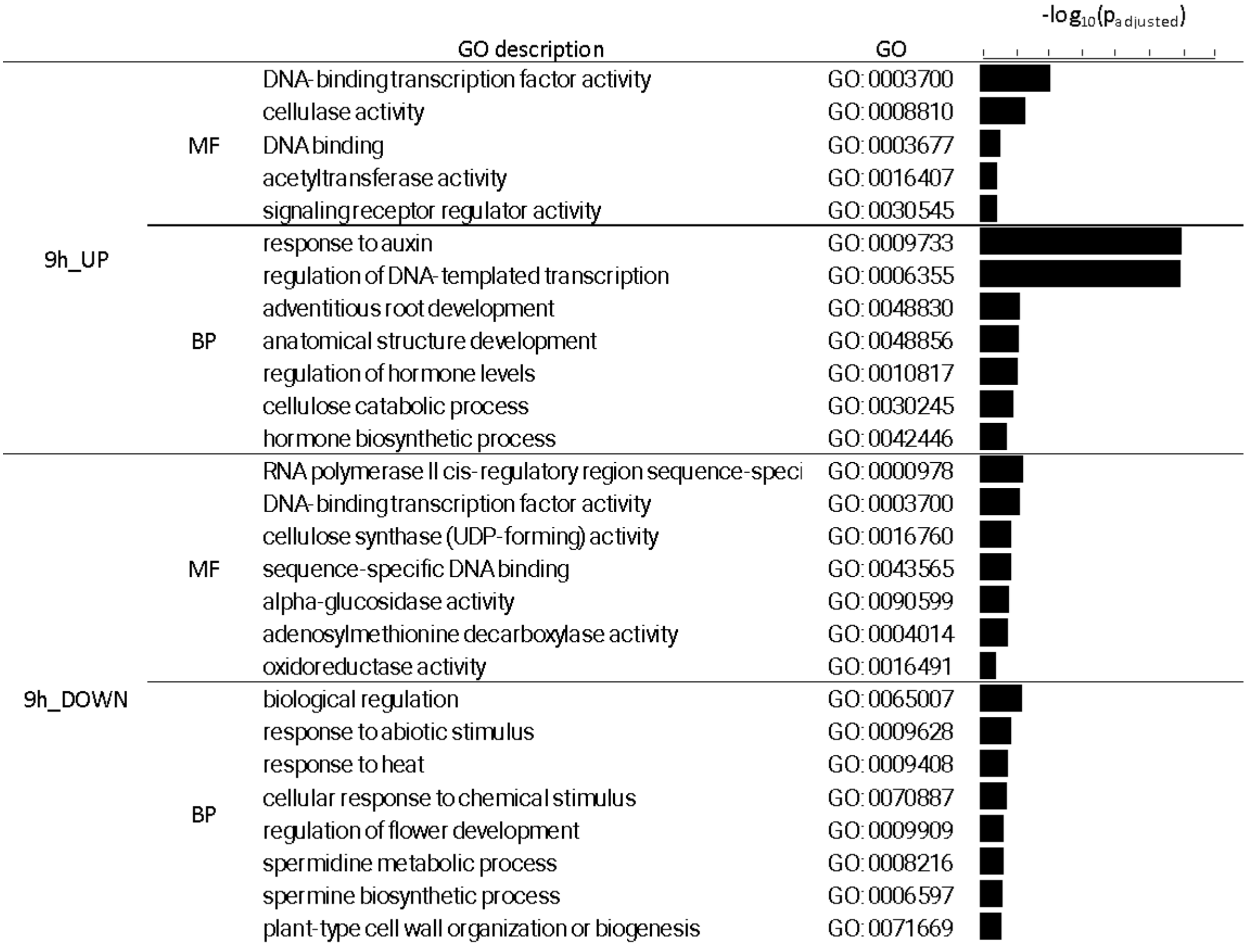

Figure S6. GO term enrichment for differentially expressed genes (DEGs) identified identified in the stem base of barley at 9 h following auxin treatment. Functional enrichment analyses of GO terms was performed by g:Profiler, using the feature for driver terms selection (Kolberg et al. 2023). 9h_UP, DEGs up-regulated at 9 h; 9h_DOWN, DEGs down-regulated at 9 h; MF, molecular function; BP, biological process.

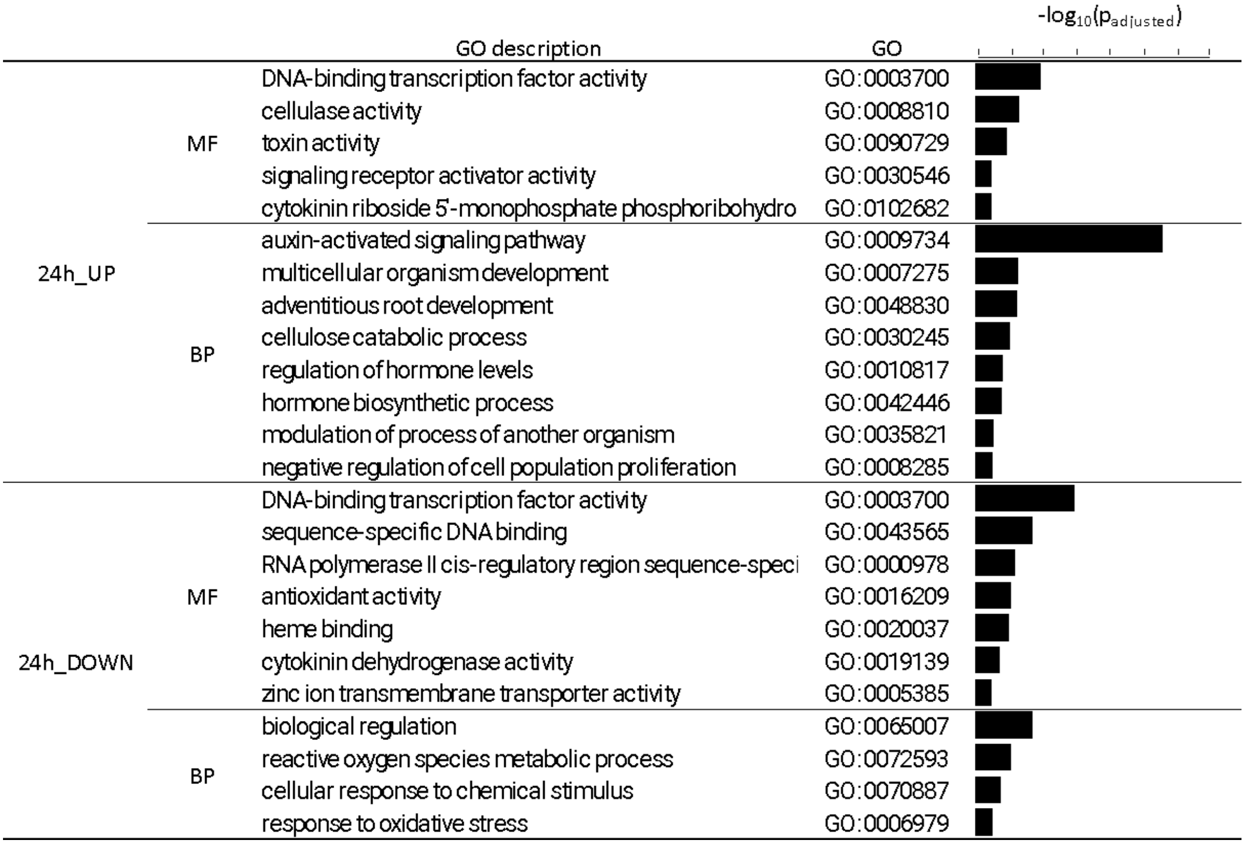

Figure S7. GO term enrichment for differentially expressed genes (DEGs) identified identified in the stem base of barley at 24 h following auxin treatment. Functional enrichment analyses of GO terms was performed by g:Profiler, using the feature for driver terms selection (Kolberg et al. 2023). 24h_UP, DEGs up-regulated at 24 h; 24h_DOWN, DEGs down-regulated at 24 h; MF, molecular function; BP, biological process.

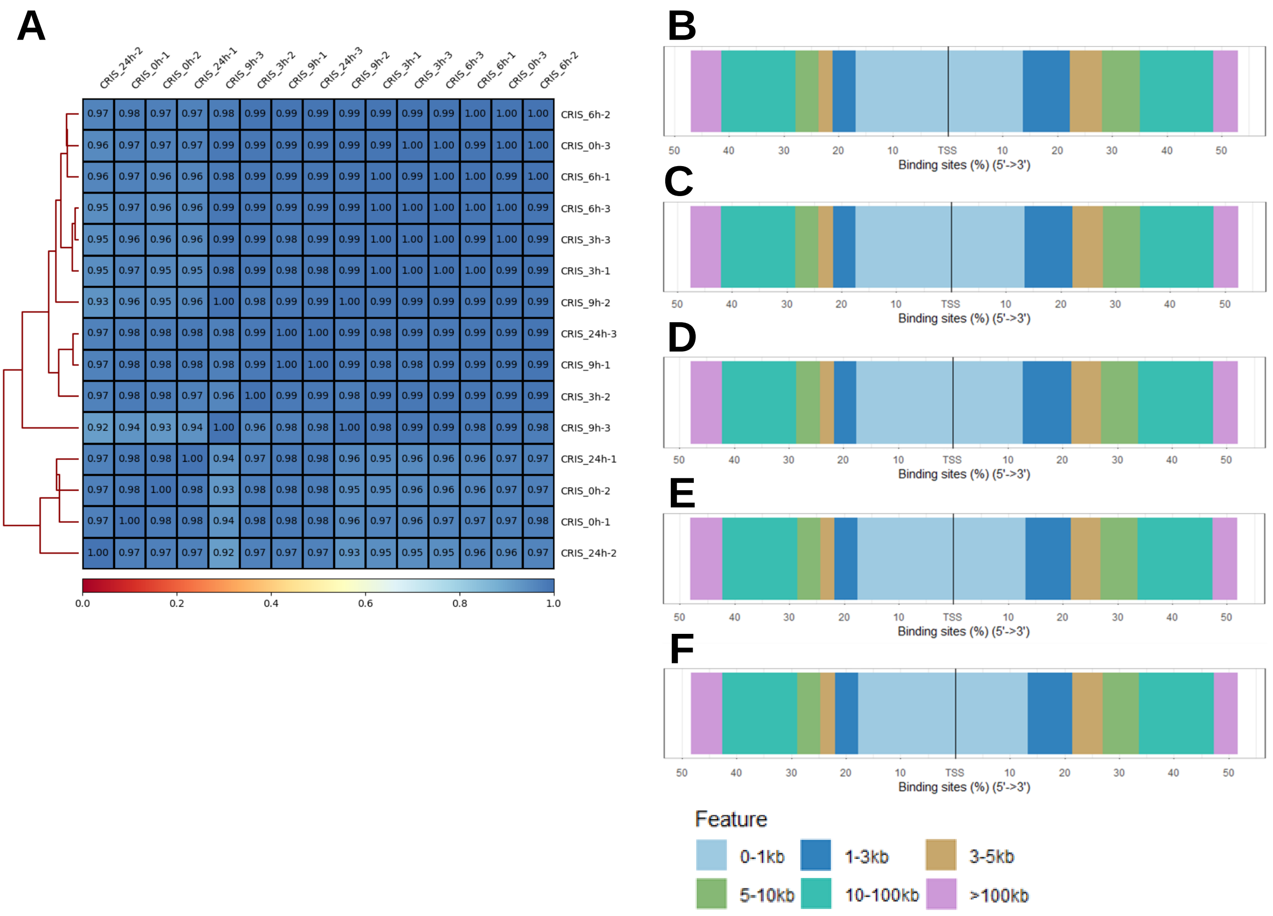

Figure S8. Quality control of ATAC-seq data. (A) Pearson pairwise correlation between ATAC-seq replicates, generated by deeptools MultiBamSummary (Ramírez et al. 2016). (B-F) Distance distribution of ATAC-seq peaks relative to transcription start site. Analysis was performed with ATAC-seq peaks identified at 0 h (B), and at 3 h (C), 6 h (D), 9 h (E), 24 h (F) following auxin treatment, analyzed by ChIPseeker (Yu et al. 2015; Wang et al. 2022).

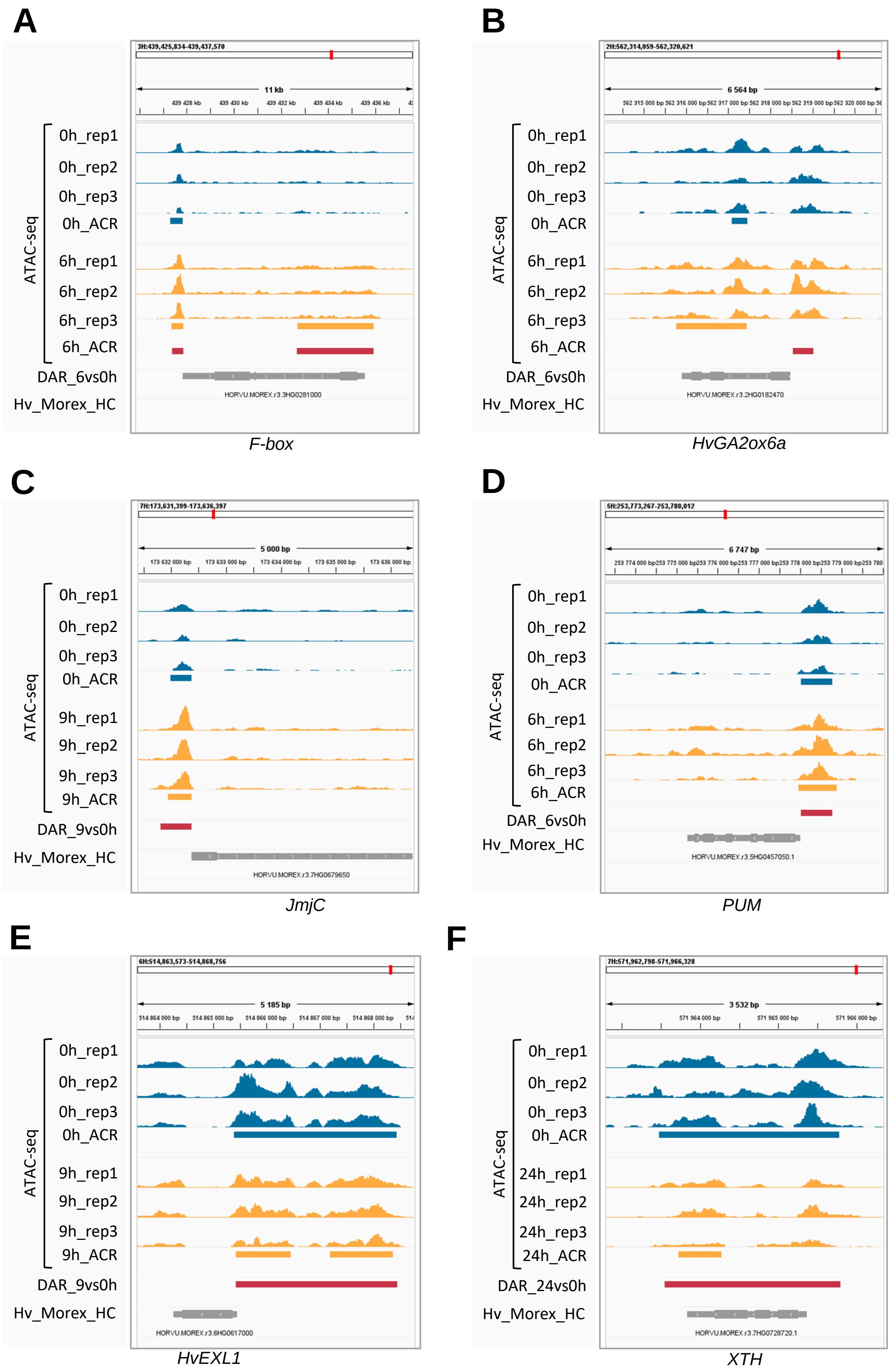

Figure S9. Examples of Differentially Accessible Regions (DARs) near genes potentially involved in crown root initiation. (A-F) Position of DARs in the genome relative to differentially expressed genes. ATAC-seq signal distribution is shown for 0 h (blue) and either 3 h, 6 h, 9 h or 24 h after auxin treatment (orange). Rectangles represent Accessible Chromatin Regions (ACRs) at 0 h (blue) and either 3 h, 6 h, 9 h or 24 h (orange), DARs (red), or genes (grey). Genome visualizations were generated using the Integrative Genomics Viewer (Robinson et al. 2011).

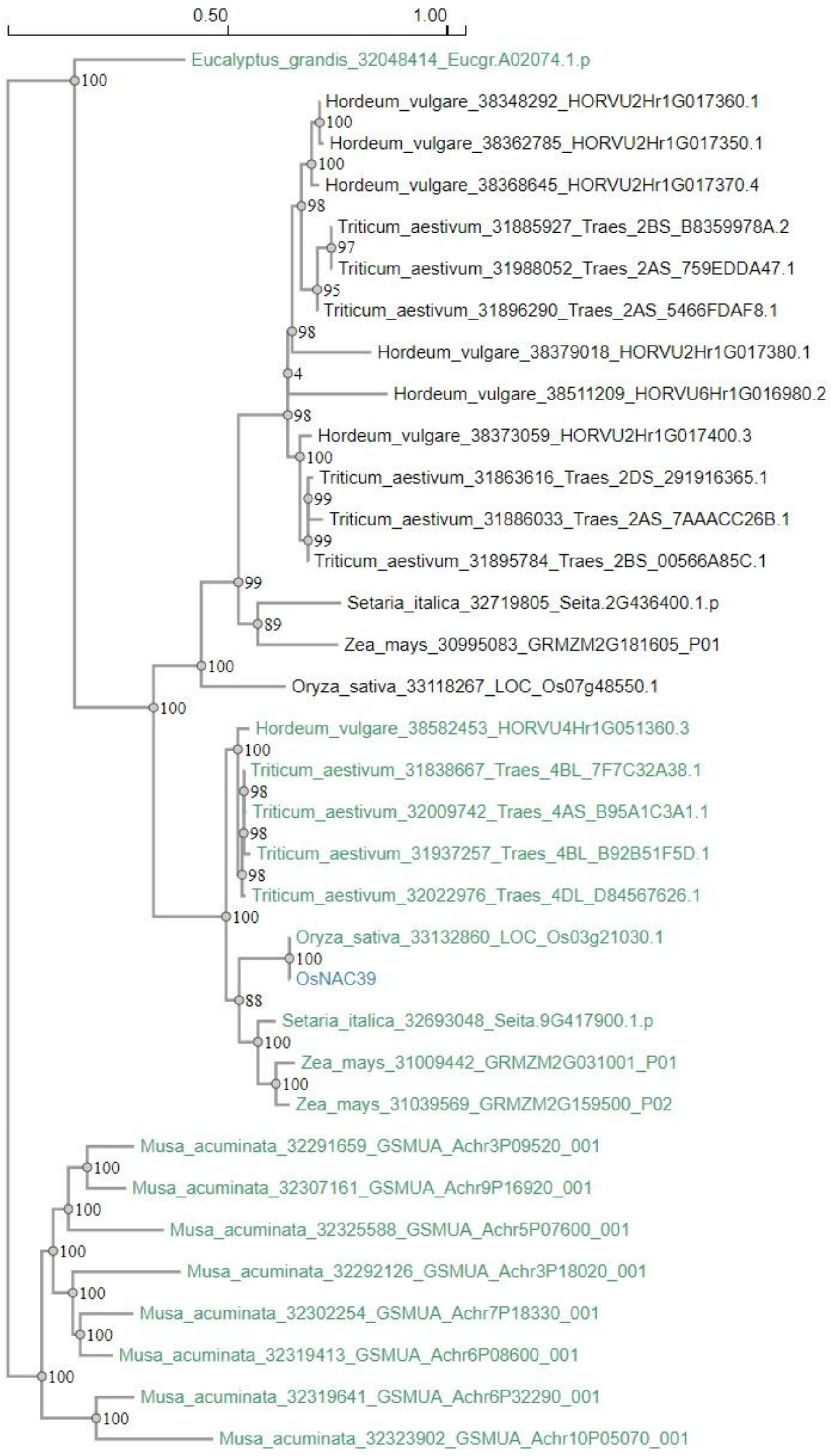

Figure S10. Identification of the barley ortholog of OsNAC39. Protein sequence of OsNAC39 was used for the identification of putative orthologs in plants by SHOOT (Emms and Kelly 2022). Predicted orthologs are marked in green.

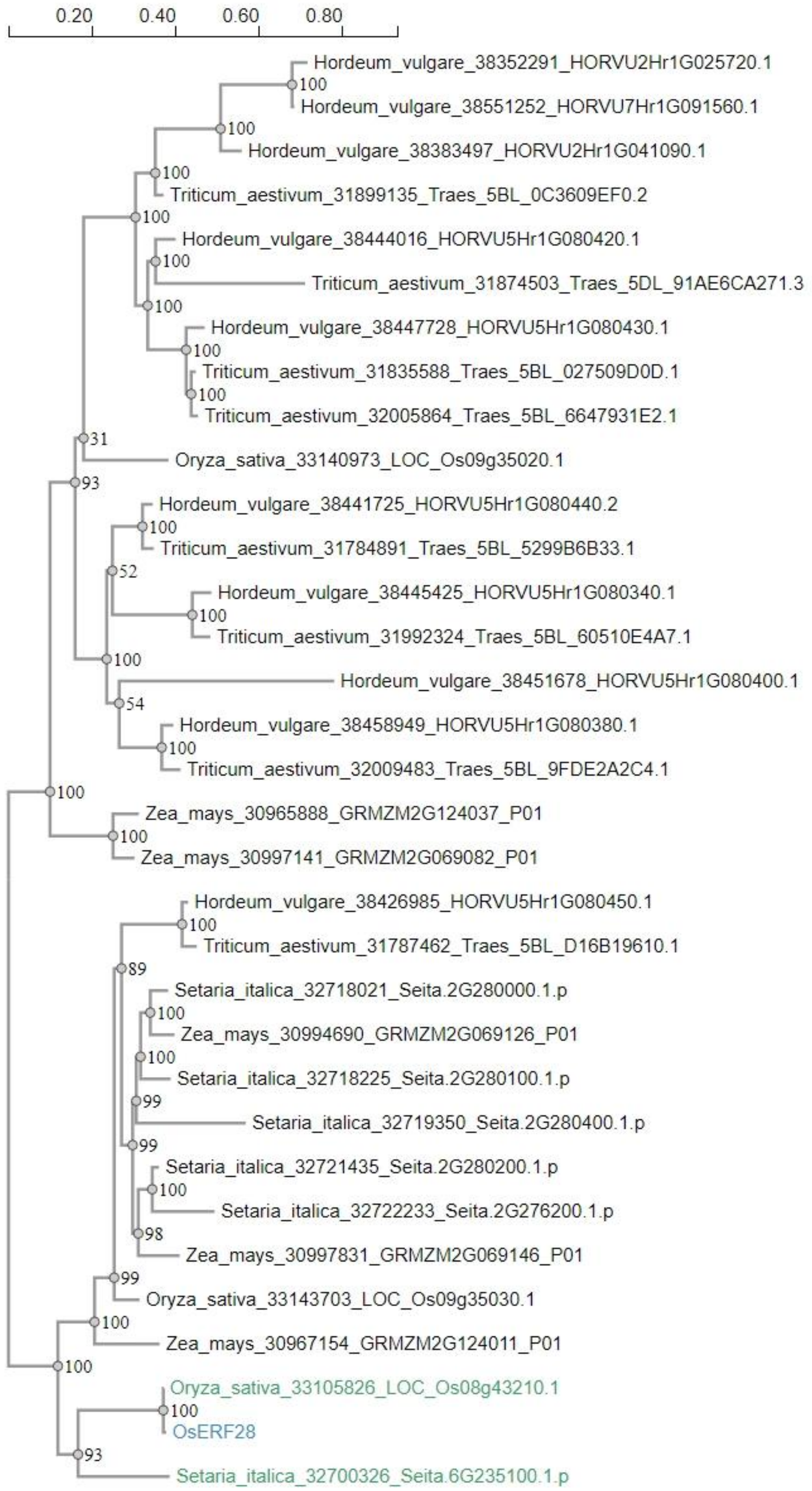

Figure S11. Identification of the barley ortholog of OsERF28. Protein sequence of OsERF28 was used for the identification of putative orthologs in plants by SHOOT (Emms and Kelly 2022). Predicted orthologs are marked in green.

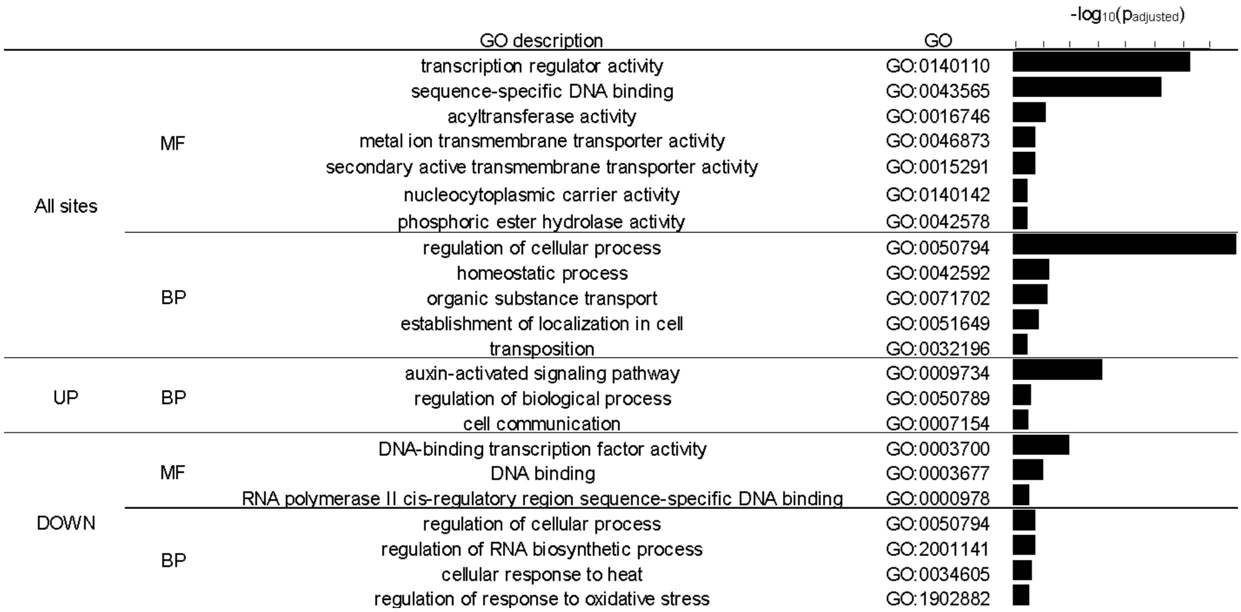

Figure S12. GO term enrichment for genes annotated to HvNAC013 binding sites. Functional enrichment analyses of GO terms was performed by g:Profiler, using the feature for driver terms selection (Kolberg et al. 2023). All sites, analysis performed with genes annotated to the complete list of HvNAC013 binding sites; UP, analysis performed with differentially expressed genes (DEGs) up-regulated at 3 h that were annotated to HvNAC013 binding sites; DOWN, analysis performed with DEGs down-regulated at 3 h that were annotated to HvNAC013 binding sites; MF, molecular function; BP, biological process.

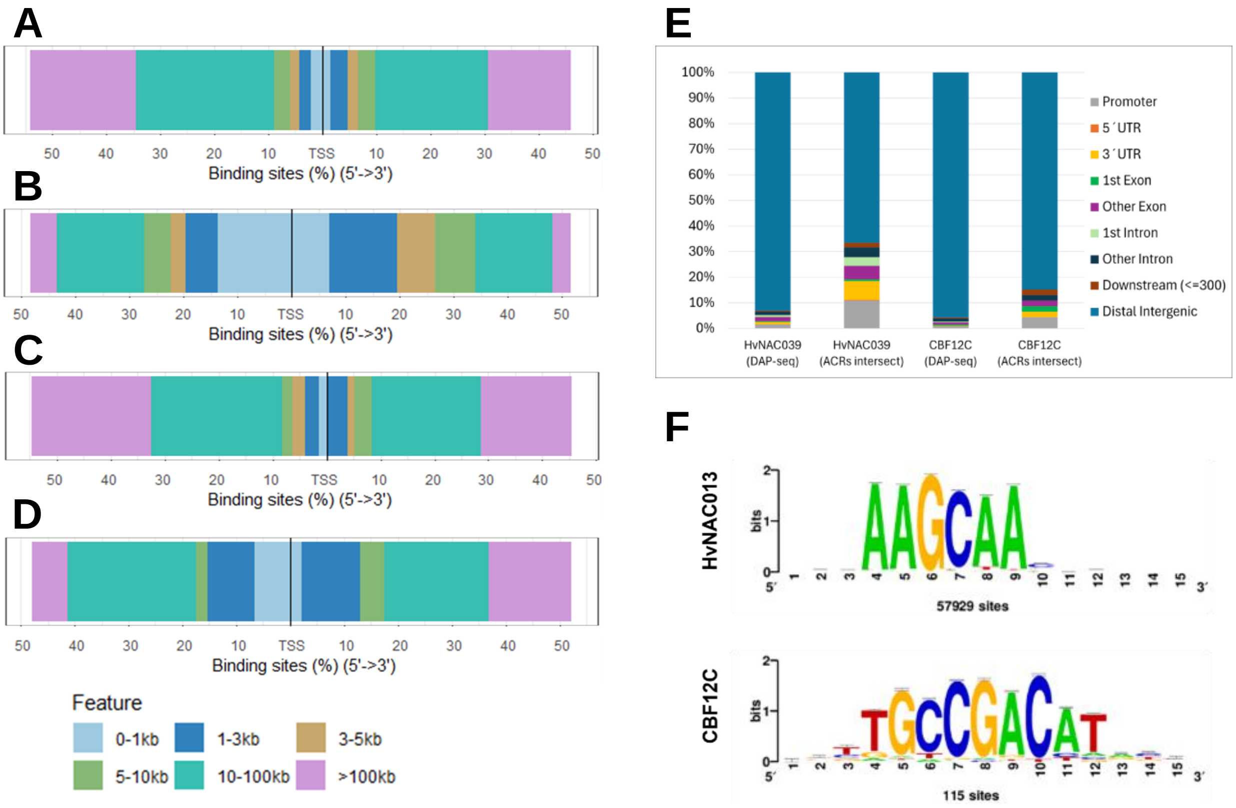

Figure S13. Comparison of a set of all DAP-seq peaks and a set of DAP-seq peaks present in Accessible Chromatin Regions (ACRs). (A-D) Distance distribution of all DAP-seq peaks (A, C) or DAP-seq peaks in ACRs (B, D) of HvNAC013 (A, B) and CBF12C (C, D) relative to transcription start site by ChIPseeker (Yu et al. 2015; Wang et al. 2022). (E) Distribution of DAP-seq peaks or DAP-seq peaks in ACRs of HvNAC013 and CBF12C relative to gene features by ChIPseeker. (F) HvNAC013 and CBF12C DNA-binding motifs derived from the set of all DAP-seq peaks.

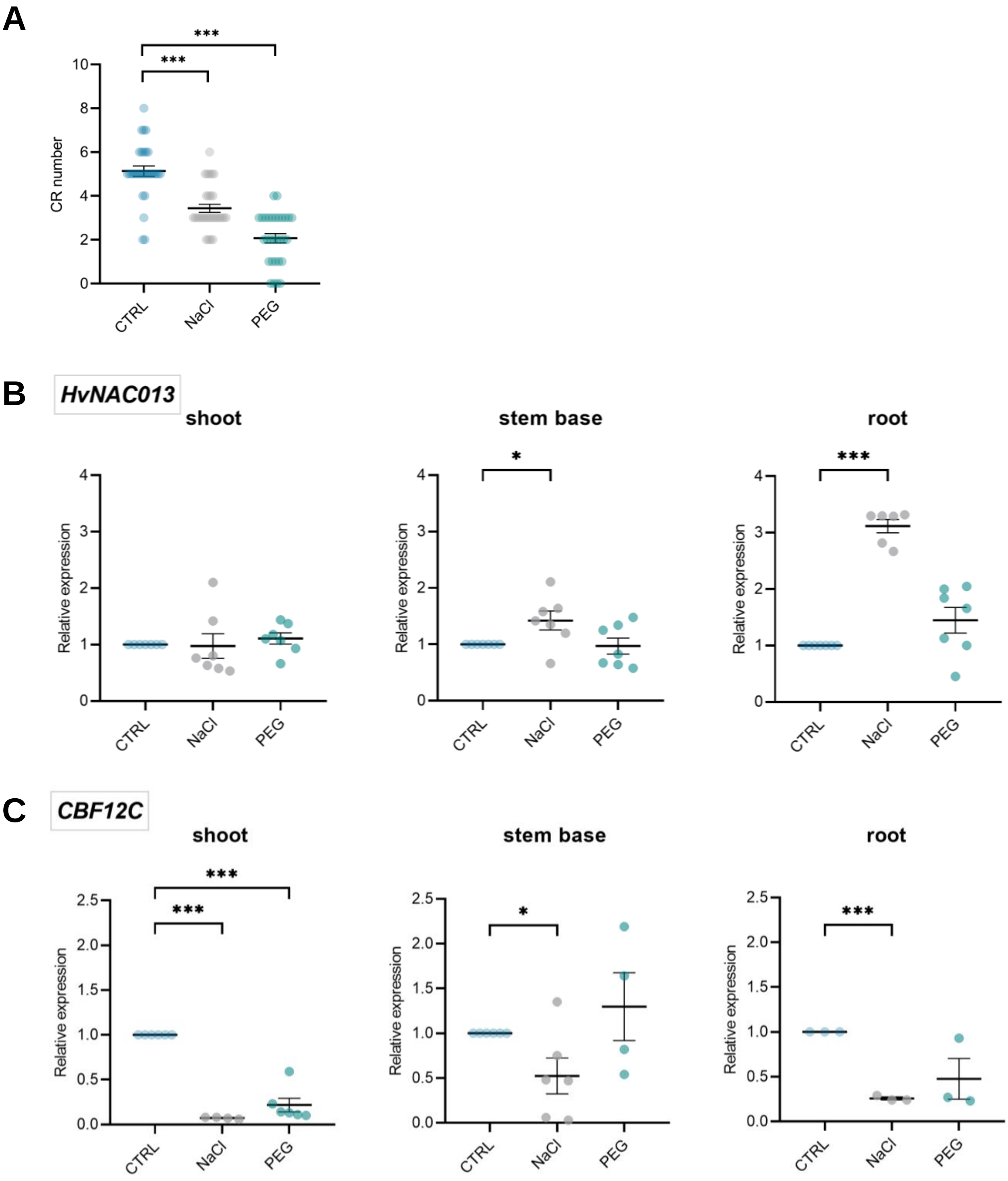

Figure S14. Expression of *HvNAC013* and *CBF12C* under stress conditions. A) Crown root number in control, salt stress and water-deficit conditions. Presented data show the mean ± standard error of the mean (SEM), n = 30. Statistical significance was supported by *t*-test. (B-C) Relative quantification of *HvNAC013* (B) and *CBF12C* (C) in barley stem base under salt and osmotic stress. Gene expression was determined relative to the control sample. Presented data show the mean ± SEM of 6 to 7 biological replicates (*HvNAC013*), or 3 to 6 biological replicates (*CBF12C*). Statistical significance was supported by *t*-test, * $p < 0.05$, ** $p < 0.01$, *** $p < 0.001$.
