# Supplementary material for: Temporal regulation of gene expression during auxin-triggered crown root formation in barley: an integrated approach
Source: Plant Cell Physiol. 2025 Jul 13;66(9):1284–303. doi: 10.1093/pcp/pcaf077 (PMC12461856; doi:10.1093/pcp/pcaf077)
Supplement: pcp-2025-e-00050-File022_pcaf077 [file pcp-2025-e-00050-file022_pcaf077.pdf]

Table S11A HvNAC013 DNA-binding motifs identified from the set of DAP-seq peaks in the Accessible Chromatin Regions.

Motifs were identified by RSAT Plants oligo analysis tool (Thomas-Chollier et al. 2012).

Best binding motif

Motif logo	Motif logo rc	Consensus sequence	Best matches (footprintDB)
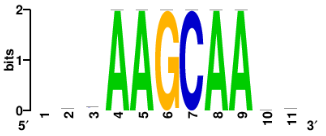	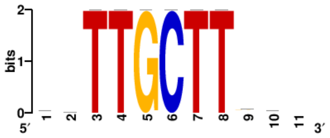	-acAAGCAAcc	NAC007 (*A. thaliana*), SMB (*A. thaliana*), NAC101 (*A. thaliana*)

Alternative binding motifs

Motif logo	Motif logo rc	Consensus sequence	Best matches
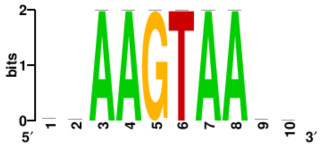	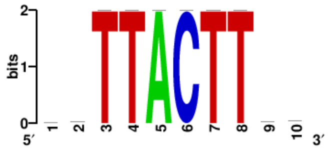	acAAGTAAaa	NAC096 (*A. thaliana*), NAC057 (*A. thaliana*), NAC017 (*A. thaliana*)
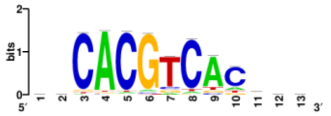	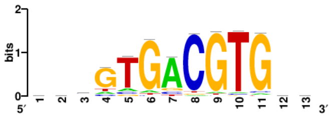	taCACGTCACgtc	BZIP63 (*A. thaliana*), bZIP44 (*A. thaliana*)
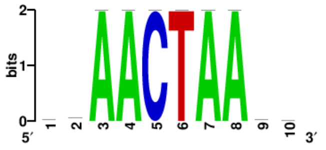	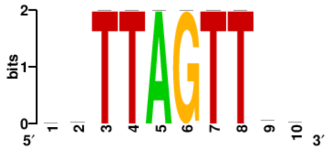	taAACTAAat	MYB52 (*A. thaliana*), ATHB23 (*A. thaliana*), MYB113 (*A. thaliana*)
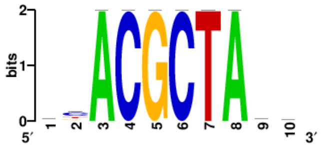	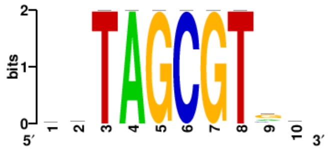	acACGCTAcc	ANAC100 (*A. thaliana*), NAM protein T128350_1.02 (*Arabidopsis lyrata*), NAM protein T129214_1.02 (*Physcomitrella patens*)
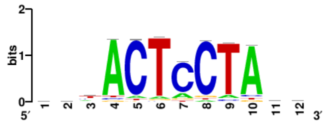	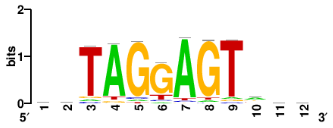	actACTCCTAct	MYB99 (*A. thaliana*), MYB-like T125588_1.02 (*Ostreococcus tauri*), ZAT (*A. thaliana*), RHL41 (*A. thaliana*)

Table S11B HvNAC013 DNA-binding motifs identified from the set of all DAP-seq peaks.

Motifs were identified by RSAT Plants oligo analysis tool (Thomas-Chollier et al. 2012).

Best binding motif

Motif logo	Motif logo rc	Consensus sequence	Best matches
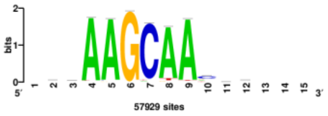	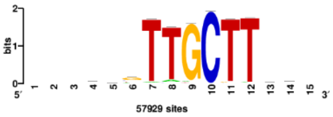	-acAAGCAAcca---	ANAC076 (*A. thaliana*),<br>NAC007 (*A. thaliana*),<br>NAC105 (*A. thaliana*)

Alternative binding motifs

Motif logo	Motif logo rc	Consensus sequence	Best matches
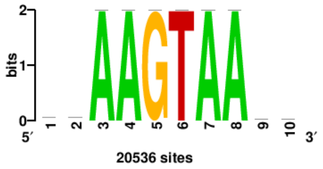	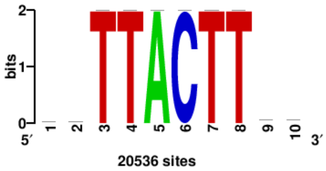	acAAGTAAaa	NAC096 (*A. thaliana*),<br>NAC057 (*A. thaliana*),<br>NAC017 (*A. thaliana*)
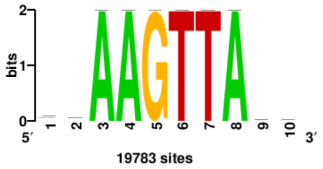	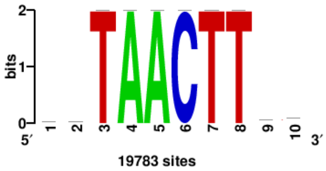	acAAGTTAtc	NAC103 (*A. thaliana*),<br>AtMYB63 (*A. thaliana*),<br>AtMYB4 (*A. thaliana*)
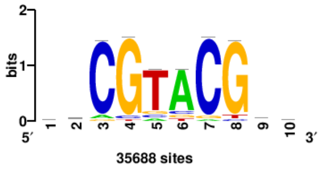	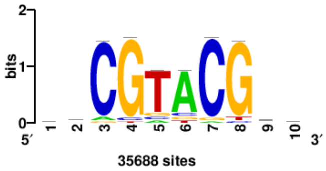	taCGTACGta	SPL1 (*A. thaliana*),<br>AT1G76580 (*A. thaliana*)
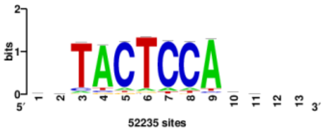	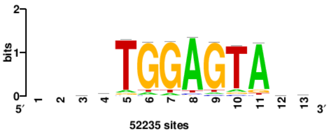	taTACTCCAtc--	At3g60580 (*A. thaliana*),<br>ZAT9 (*A. thaliana*),<br>AT5G04390 (*A. thaliana*)
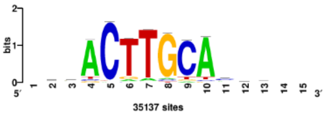	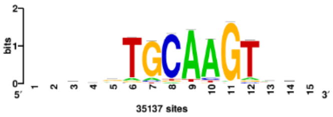	-ttACTTGCAcaa—	FUS3 (*A. thaliana*)<br>NAC017 (*A. thaliana*)<br>bHLH80 (*A. thaliana*)

<u>Table S11C CBF12C DNA-binding motifs identified from the set of DAP-seq peaks in the Accessible Chromatin Regions.</u>

Motifs were identified by RSAT Plants oligo analysis tool (Thomas-Chollier et al. 2012).

Best binding motif

**Motif logo**	**Motif logo rc**	**Consensus sequence**	**Best matches**
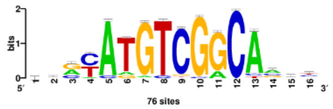	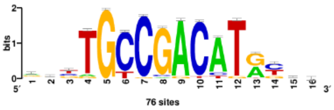	atgcATGTCGGCAact	DREB1A (*A. thaliana*), DREB1F (*A. thaliana*), HRD (*A. thaliana*), DREB1C (*A. thaliana*), ERF027 (*A. thaliana*), DREB1B (*A. thaliana*), AT1G12630 (*A. thaliana*)

<u>Table S11D CBF12C DNA-binding motifs identified from the set of all DAP-seq peaks.</u>

Motifs were identified by RSAT Plants oligo analysis tool (Thomas-Chollier et al. 2012).

Best binding motif

**Motif logo**	**Motif logo rc**	**Consensus sequence**	**Best matches**
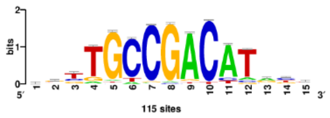	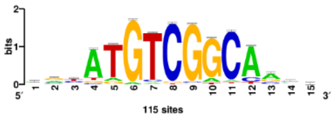	agtTGCCGACATact	CBF2 (*A. thaliana*), CBF4 (*A. thaliana*), DREB1B (*A. thaliana*), DREB1C (*A. thaliana*), ERF027 (*A. thaliana*)
