# Supplementary material for: Temporal regulation of gene expression during auxin-triggered crown root formation in barley: an integrated approach
Source: Plant Cell Physiol. 2025 Jul 13;66(9):1284–303. doi: 10.1093/pcp/pcaf077 (PMC12461856; doi:10.1093/pcp/pcaf077)
Supplement: pcp-2025-e-00050-File023_pcaf077 [file pcp-2025-e-00050-file023_pcaf077.pdf]

Table S12 List of primers and probes used in this study.

Gene name		Sequence (5´-3´)
EF2α HORVU.MOREX.r3.5HG0529120.1	Fw	CAGCGTACTGTTATCTGGATGG
	Rev	CAGGGTTGCATTCTTTGTGATG
	Probe	[FAM]CACTGTTGCGTTGGTTGGTTTGGA[BHQ1]
ACT HORVU.MOREX.r3.1HG0003140.1	Fw	GGATCTCACGGACTCCCTTAT
	Rev	GCTTCTCCTTGATGTCCCTTAC
	Probe	[FAM]TCACCGAGAGAGGTTACTCCTTCACA[BHQ1]
ENT HORVU.MOREX.r3.5HG0490950.1	Fw	GACCAAGAGGACGATCTTTCAG
	Rev	ACCAAATGTACTTCGCTCTCC
	Probe	[FAM]ACCGTTTCCTTATGCAAGGCCACA[BHQ1]
CBF12C HORVU.MOREX.r3.5HG0497580.1	Fw	TTCAGGTGCCGGCTTTCTCC
	Rev	ATTCTCCGTTCTCCCAGTGC
	Probe	[FAM]CGTACTACGCGGGCTTTGCCCAGGGGATGC[TAM]
HvNAC013 HORVU.MOREX.r3.4HG0379700.1	Fw	CAAGGACCGCAAGTACCCGAC
	Rev	AGCGTCTTCTTCATGCCGAC
	Probe	[FAM]GGCGACGGAGTCCGGCTACTGGAAGGCCA[BHQ1]
CNG HORVU.MOREX.r3.6HG0583650.1	Fw	GGGCTGATGACCTGAAGTTT
	Rev	GGTTCTCCACTGTTGTGAGTAG
	Probe	[FAM]ACTCCATAGCAAACAGCTCCAGCA[BHQ1]
